# A robust and versatile mass spectrometry platform for comprehensive assessment of the thiol redox metabolome

**DOI:** 10.1016/j.redox.2018.02.012

**Published:** 2018-02-19

**Authors:** T.R. Sutton, M. Minnion, F. Barbarino, G. Koster, B.O. Fernandez, A.F. Cumpstey, P. Wischmann, M. Madhani, M.P. Frenneaux, A.D. Postle, M.M. Cortese-Krott, M. Feelisch

**Affiliations:** a Clinical and Experimental Sciences, Faculty of Medicine, University of Southampton; NIHR Southampton Biomedical Research Centre, University of Southampton and University Hospital Southampton NHS Foundation Trust, Southampton, UK; b Cardiovascular Research Laboratory, Division of Cardiology, Pulmonology & Vascular Medicine, Medical Faculty, Heinrich Heine University, Düsseldorf, Germany; c Institute of Cardiovascular Sciences, University of Birmingham, Birmingham, UK; d Norwich Medical School, University of East Anglia, Norwich, UK

**Keywords:** BSA, bovine serum albumin, Cys, cysteine, CysSS, cystine (cysteine disulfide), CysGly, cysteinylglycine, GluCys, glutamylcysteine, GSH, glutathione (reduced), GSSG, glutathione disulfide (oxidized), HPLC, high pressure liquid chromatography, HCysSS, homocystine (homocysteine disulfide), Hcys, homocysteine, LOD, limit of detection, LOQ, limit of quantification, LC-MS/MS, liquid chromatography tandem mass spectrometry, LMW, low molecular weight, mBB, monobromobimane, MRM, multiple reaction monitoring, NEM, N-etylmaleimide, Nrf2, nuclear factor erythroid2-related factor 2, ROS, reactive oxygen species, RSI, reactive species interactome, RSS, reactive sulfur species, RT, room temperature, H_2_S, sulfide (H_2_S+HS^−^+S^2^^−^), UPLC, ultra high performance liquid chromatography, Oxidative stress, Redox status, Reactive species interactome, Glutathione, Hydrogen sulfide, Persulfides, Thiol-maleimide michael addition

## Abstract

Several diseases are associated with perturbations in redox signaling and aberrant hydrogen sulfide metabolism, and numerous analytical methods exist for the measurement of the sulfur-containing species affected. However, uncertainty remains about their concentrations and speciation in cells/biofluids, perhaps in part due to differences in sample processing and detection principles. Using ultrahigh-performance liquid chromatography in combination with electrospray-ionization tandem mass spectrometry we here outline a specific and sensitive platform for the simultaneous measurement of 12 analytes, including total and free thiols, their disulfides and sulfide in complex biological matrices such as blood, saliva and urine. Total assay run time is < 10 min, enabling high-throughput analysis. Enhanced sensitivity and avoidance of artifactual thiol oxidation is achieved by taking advantage of the rapid reaction of sulfhydryl groups with N-ethylmaleimide. We optimized the analytical procedure for detection and separation conditions, linearity and precision including three stable isotope labelled standards. Its versatility for future more comprehensive coverage of the thiol redox metabolome was demonstrated by implementing additional analytes such as methanethiol, N-acetylcysteine, and coenzyme A. Apparent plasma sulfide concentrations were found to vary substantially with sample pretreatment and nature of the alkylating agent. In addition to protein binding in the form of mixed disulfides (S-thiolation) a significant fraction of aminothiols and sulfide appears to be also non-covalently associated with proteins. Methodological accuracy was tested by comparing the plasma redox status of 10 healthy human volunteers to a well-established protocol optimized for reduced/oxidized glutathione. In a proof-of-principle study a deeper analysis of the thiol redox metabolome including free reduced/oxidized as well as bound thiols and sulfide was performed. Additional determination of acid-labile sulfide/thiols was demonstrated in human blood cells, urine and saliva. Using this simplified mass spectrometry-based workflow the thiol redox metabolome can be determined in samples from clinical and translational studies, providing a novel prognostic/diagnostic platform for patient stratification, drug monitoring, and identification of new therapeutic approaches in redox diseases.

## Highlights

• The thiol redox metabolome comprises free and bound thiols, disulfides, and sulfide.
• We developed a LC-MS/MS platform for detection of the thiol redox metabolome in biological samples.
• Thiols in blood, urine and saliva are stabilized by derivatization with N-ethylmaleimide, also preventing oxidation.
• Total and acid-labile thiols are determined following addition of DTT and acid.
• Accuracy was investigated by comparison with an established method to assess GSH and GSSG.
• Applicability was shown for plasma of 10 healthy volunteers, blood cells, urine and saliva.

## 1 Introduction

Many biological processes that have previously been associated with an overproduction of reactive oxygen species (ROS) and/or an impaired antioxidant and free radical scavenging capacity were thought to culminate in ‘oxidative stress’, cell death and tissue damage. More recently, such conditions are interpreted to reflect situations in which a shift in redox poise has occurred, affecting both global and regional redox status and cysteine-based redox signaling [1], [2], [3]. Importantly, unfavorable outcomes may not be inevitable if intervened early on. Consequently, there is an increased interest in analytical methods that provide a more refined mapping of the associated metabolic changes and enable further study of the underlying mechanisms.

In parallel with those developments, there has been a resurgence of interest in the biological effects of hydrogen sulfide (H_2_S)^2^ and related oxidation products such as persulfides and polysulfides [4], [5], [6]. No specific biological targets have yet been identified for those compounds, and many of their effects appear to be mediated by interaction with metals, heme proteins or redox interaction with other sulfur species. Moreover, sulfide is involved in post-translational protein modification (persulfidation, S-sulfhydration) whereby reactive cysteine groups involved in redox signaling are modified, resulting in altered chemical reactivity and protein function [7].

Interestingly, ROS were shown to chemically and functionally interact with nitric oxide (NO) and other reactive nitrogen species (RNS), as well as with sulfide-related reactive sulfur species (RSS) such as thiyl radicals, persulfides and polysulfides. Our group recently proposed that the interaction of ROS, RNS and RSS with each other and with their thiol targets constitute a ‘reactive species interactome’ (RSI) that regulates fundamental cellular processes involved in stress signaling [3]. The RSI enables single cells and whole organisms to sense and adapt to alterations in nutritional, metabolic and environmental conditions [3]. A corollary of this concept is that assessment of only a few constituents of this RSI as “markers of oxidative stress” (e.g. oxidized lipids) will not be sufficient to provide useful insight as they cannot adequately capture the complexity of the chemical interactions within the RSI. This may explain why the redox community has been struggling in successfully identifying the key regulatory nodes and operating principles of this interaction network, and to e.g. understand how specific thiols and redox switches like the nuclear factor erythroid 2 [NF-E2]-related factor 2 (Nrf2) contribute to redox homeostasis in health and disease. Thus, the renewed interest in sulfide related phenomena, antioxidant processes and redox signaling demands quantification of pertinent redox-regulated events in much higher resolution than hitherto accomplished.

Given that glutathione is the major intracellular low-molecular-weight (LMW) aminothiol, much of the earlier ‘oxidative stress’ literature revolved around pathways of formation and degradation of this ubiquitous and (according to Helmut Sies) “inevitable” antioxidant [8], besides the measurement of protein, lipid and DNA oxidation products as biomarkers of an increased oxidative burden. As a result, many different methods have been developed to quantify glutathione and related metabolites in blood and tissues [9], [10], [11], [12], and the ratio of reduced over oxidized glutathione (GSH/GSSG) has been used for decades as a sensitive oxidative stress biomarker [11], [13], [14]. In many of these methods the sulfhydryl (-SH) group of the aminothiol is derivatized with an electrophile such as monobromobimane (mBB), iodoacetic acid (IAA), iodoacetamide (IAM) or N-ethylmaleimide (NEM). This reaction step serves two purposes: i) it prevents ambient air mediated oxidation of the thiols to their corresponding disulfides [10], [15] and ii) it conveniently forms a fluorescent product [16], often improving sensitivity limits for detection.

More recently, the cysteine/cystine (Cys/CysSS) redox couple emerged, along with GSH/GSSG, as a powerful predictor of cardiovascular mortality [17]. Circulating total homocysteine has been used as an integrative biomarker of folate and methionine metabolism, cobalamine deficiency and cardiometabolic risk for some time [18], while the mixture of “*free thiols”* and “*protein-bound thiols”* (including glutathione, cysteine, and homocysteine; please refer to Box 1 for definitions of terms in italics) was proposed to reflect the plasma redox thiol status more than 2 decades ago [19], [20]. Most of the methods used to determine these species employ either high pressure liquid chromatography (HPLC) or a similar chromatographic separation technique coupled to a ultraviolet, fluorescence or electrochemical detector. In more recent applications these detectors have been replaced by mass spectrometers, potentially offering more specific and sensitive measurements [21], [22].

Box 1 **The Thiol Redox Metabolome.**

*Thiol,* compound carrying a sulfhydryl (–SH) group (often from a cysteine).

According to their **molecular weight** they are generally classified in (a) *low molecular weight* (LMW) thiols (e.g. cysteine, homocysteine, glutathione) and (b) *high molecular weight* thiols, which include protein thiols.

According to their **redox state** they are classified as (a) *reduced or “free” thiols (RSH)*, and (b) oxidized or “bound” thiols, where a thiol is bound to another thiol via a disulfide bridge including (b1) *symmetric disulfides (RSSR)*, where a disulfide bond links two equal molecules e.g. 2 cysteines to yield cystine, 2 glutathione to give GSSG etc., or (c) *mixed or asymmetric disulfides (RS-SP)*, carrying a disulfide bond between two different LMW thiols or a LMW thiol and a protein.

According to the **measurement protocol** thiols are also classified in:

*(a) Free thiols,* the concentration of thiols that are assessed in a specimen by derivatization with a thiol reactive reagent, like NEM, mBB, or IAM, for example. It may comprise (a1) LMW free thiols and (a2) (if proteins are not removed from the specimen before derivatization) protein free thiols + LMW thiols (which as sum are defined as *total free thiols)*.
*(b) Total thiols (or DTT-reactive thiols),* the concentration of all thiols that can be determined if a specimen is treated with a strong reducing agent such as DTT, which reduces disulfide bonds and liberates all oxidized bound thiols (RSSR, RSSP), and comprises *free thiols (RSH)* + *oxidized* / *bound thiols (RSSR, RSSP)*.
*(c) Acid-labile thiols*, the concentration of LMW thiols that is liberated from binding to proteins or sulfane sulfur compounds following the treatment of a biological sample with mineral acid (e.g. HCl); in the case of sulfide, this is known as “acid-labile sulfur” equivalents and originates from the decomposition of inorganic and organic persulfides and polysulfides.

According to their **concentrations**, the thiols define:

(a) the *redox status* of a tissue/cell, according to the ratio of reduced/oxidized thiols
(b) the *redox reserve* (or total thiol status), which is an index of the total reducing capacity of a cell/tissue or organism that defines its resilience to oxidative modification.

There is accumulating evidence that these mesurements may be applied in clinical settings to predict morbidity and mortality from redox diseases [3]. Interestingly, even the rather simple spectrophotometric measurement of total free thiol availability using a non-selective free sulfhydryl probe (Ellman's reagent) in plasma or serum has been demonstrated to have an astonishing power to predict graft failure and mortality in renal transplant patients and cardiovascular mortality in a heart failure cohort [23], [24]. This suggests that the single free SH group of circulating serum albumin may be another integrative biomarker of redox-sensitive events in vivo [3]. S-cysteinylated and S-glutathionylated albumin have also been proposed to represent useful biomarkers of oxidative stress [25], [26], and albumin itself may be an important transporter of low-molecular weight thiols by allowing the formation of reversible mixed disulfides. The complexity of the methionine recycling, transsulfuration and glutathione metabolic pathways suggests that no single biomarker will adequately capture overall metabolic and redox status of all of these pathways at a global level.

Many different assays for the quantification of sulfide in seawater or simple aqueous buffer systems exist, but not all are suited for the detection of sulfide in biological material [27], [28]. Modifications of a century-old colorimetric technique, the methylene blue assay [29], [30], have been used by many research groups to detect sulfide in blood, and reports from several groups hinted at associations between blood pressure or metabolic status and methylene blue reactive material (interpreted to reflect sulfide levels). While the overall specificity of the methylene blue assay for sulfur and its low running costs continue to contribute to its popularity, doubts have been raised as to its reliability for the quantification of sulfide in complex biological media [31]. In the last couple of years a large number of fluorimetric probes have been developed to detect sulfide [32], but many retain some cross-reactivity with other RSS. Moreover, an HPLC-based method with fluorimetric detection that employs the reaction of sulfide with mBB has been developed [33], [34], [35] and is now widely used for sulfide quantification in biological material. Most recently, the same reaction principle was exploited to develop a more specific detection technique for sulfide using liquid chromatography hyphenated to tandem mass spectrometry [36].

In spite of all these developments, still relatively little is known about specific pathways of sulfide metabolism (other than its oxidation to thiosulfate and sulfate) in humans, and/or how changes in circulating sulfide concentrations relate to plasma thiol redox status. Some of the electrophilic compounds used to derivatise aminothiols before chromatographic separation, such as mBB [16] and NEM [37], [38], are known to also react with sulfide. Surprisingly, this potential does not yet seem to have been exploited in more recent approaches using mass spectrometry to measure aminothiol concentrations together with sulfide.

The aim of the present study was to describe the development of an analytical platform that allows simultaneous determination of the thiol redox metabolome, including *total* and *free thiols*, and their corresponding disulfides as well as sulfide in complex biological matrices such as human blood, saliva and urine. We here present first proof-of-concept results of our own method development using NEM as alkylating agent for thiols and sulfide to achieve this objective, and discuss advantages, pitfalls and limitations.

## 2 Materials and methods

### 2.1 Chemicals and reagents

Unless otherwise stated all reagents and materials were of the highest purity available and purchased from Sigma-Aldrich (Gillingham, UK or Munich, Germany). Dual stable isotope (^13^C, ^15^N) labelled reduced (GSH) and oxidized glutathione (GSSG) were from Cambridge Isotopes and obtained from CK Isotopes Ltd (Newtown Unthank, UK), N-ethyl-d_5_-maleimide (d_5_-NEM) was from Sigma-Aldrich. Sodium persulfide (Na_2_S_2_) was from Dojindo Europe (Neuss, Germany). Hanks´ balanced salt solution + Ca^2+^ + Mg^2+^ (HBSS^+^) was obtained from Invitrogen, bovine serum albumin (BSA) from Carl Roth (Karlsruhe, Germany) and HPLC-grade solvents from either VWR (Darmstadt, Germany) or Fisher Scientific (UK). Argon gas (> 99.99%) was from BOC Group (Guildford, UK). Ultrapure N_2_ gas was produced by a nitrogen generator (Parker Balston, UK).

### 2.2 Human participants

Blood was taken from healthy volunteers of either gender (20–58 years old) with informed consent to participate before enrollment. Procedures were approved by the ethics committees of the Heinrich Heine University of Dusseldorf (ClinicalTrials.gov Identifier: NCT02272530) and the University of Southhampton (ERGO 30507/31426) and conducted in accordance with the Declaration of Helsinki.

### 2.3 Instrumentation and chromatography

The chromatography system used in most of the studies described was a Waters Aquity ultrahigh performance liquid chromatography (UPLC) system with a thermostatted autosampler (kept at 5 °C) and an ultrahigh performance binary pump, coupled to a triple-quadrupole mass spectrometer (Xevo TQ-S, Waters) equipped with a heated electrospray ionization source (ESI).

Chromatographic separation of the target analytes was achieved using a 1.6 µm Modus 100 × 2.1 mm Aqua UPLC column (Chromatography Direct, Runcorn, UK) kept at a temperature of 30 °C; mobile phase A was H_2_O with 0.15% formic acid and 5 mM ammonium formate and mobile phase B was 95% acetonitrile with 5% H_2_O, 0.15% formic acid and 5 mM ammonium formate. The chromatographic gradient starts at 99% A, decreasing to 60% A over 4.5 min, before dropping to 0% A over 0.5 min and being held at that level for 1.5 min. The column is brought back up to 99% mobile phase A over 0.5 min and held at 99% for a further 1 min to equilibrate. A flow rate of 0.2 ml/min was used throughout, and total run time including equilibration was 8 min. An injection volume of 5 µl was used, with a wash step every ten injections consisting of 100% mobile phase B for 5 min, followed by a blank using the regular gradient to control for potential carry-over and column equilibration. We found that carry-over was negligible with two needle rinse steps (300 µl methanol, followed by 600 µl H_2_O /acetonitrile 90/10%, v/v) between injections.

Our initial separation attempts used an Aquity UPLC CSH C_18_ (1.7 µm) 2.1 × 100 mm column (Waters) kept at 30 °C; mobile phase A was H_2_O with 5 mM ammonium formate and mobile phase B was 95% acetonitrile with 5% H_2_O and 5 mM ammonium formate. The chromatographic gradient started at 95% A, decreasing to 40% A over 5 min before returning to 95% A over 1 min and being held at that level for a further 1 min, resulting in a total run time of 7 min at a constant flow rate of 0.2 ml/min. Experimental results depicted in Fig. 3 and some panels of Fig. 4 were obtained using these separation conditions, as indicated in the figure legend.

Mass spectrometry settings were as follows: capillary voltage 2.80 kV, source offset 6 V, desolvation gas flow 800 L/h, cone gas flow 150 L/h, nebulizer pressure 7.0 bar, collision gas (argon) flow 0.14 ml/min, desolvation temperature 250 °C. All analytes were detected using positive ionization in multiple reaction monitoring (MRM) mode with specific precursor/product ion combinations identified and cone and collision energies optimized for each individual compound during direct infusion of authentic standards. For this, compounds were dissolved in NEM-containing buffer at a concentration of approx. 1 µM and diluted in water containing 5 mM ammonium formate. In positive ionization mode the precursor (parent) compound is usually the protonated form of the starting material, and the fragmentation profile is dependent on the cone and collision energies applied; optimal detection parameters for these were established separately for each compound during direct infusion of authentic standards by varying those parameters and selecting the most suitable product (daughter) ion by intensity and specificity. In order to maximise sensitivity, accuracy and selectivity specific MRM time windows were used to minimize the number of concurrent MRM transitions being monitored. For quantification purposes the MRM transitions were therefore only monitored during the time window where the relevant compounds elute. For specifics including the chemical structures of analytes and *m/z* values of precursor>product couples monitored see Table 1. Signals were captured and data processed using MassLynx v.4.0 and Quanlynx v.4.0 software (Waters).

**Table 1:** Structures, MS parameters, elution times and limits of detection (LOD) and quantification (LOQ) for the 12 analytes and 3 internal standards investigated. (n.d. - not determined).

**Analyte/Internal Standard**	**Structures of Oxidized Thiols, NEM Derivatised Reduced Thiols and Sulfide**	**Precursor Ion *m/z***	**Product Ion *m/z***	**Cone Energy/V**	**Collision Energy/V**	**Elution time (C**_**18**_**)/min**	**Elution time (Aqua)/min**	**LOD/nM**	**LOQ/nM**
**Reduced Thiols (NEM Derivatized)**									
N-Acetlycysteine		289.0	201.0	17	14	n.d.	4.05	0.5	4
Coenzyme A		892.5	389.0	12	31	n.d.	n.d.	n.d.	n.d.
Cysteine		247.1	158.1	8	18	2.18	2.80	8	20
Cysteinyl-glycine		304.0	212.0	8	8	2.30	2.90	20	50
Glutamyl-cysteine		376.0	246.6	5	10	1.80	3.20	0.2	0.5
Glutathione		433.1	304.0					0.2	0.5
^**13**^**C**_**2**_,^**15**^**N** Glutathione *		436.1	307.0	6	13	2.00	3.26		
Homocysteine		261.1	56.0	8	16	2.40	3.08	1	4
**Oxidized Thiols**									
Cystine		241.0	152	8	12	1.15	1.10	60	100
Glutathione (Oxidized)		613.0	355.0					0.5	2
^**13**^**C**_**4**_,^**15**^**N**_**2**_ Glutathione (Oxidized)*		619.0	361.0	10	20	1.00	2.50		
Homocystine		269.1	136.1	14	4	1.20	1.50	4	7
**Sulfide Related Species (NEM Derivatized)**									
Sulfide		285.1	160.1					4	8
**D**_**10**_-NEM_2_-Sulfide		295.1	165.1	8	14	4.85	5.90		
Persulfide		317.0	160.1	8	14	5.50	n.d.	n.d.	n.d.
Methanethiol		173.6	74.6	1	11	n.d.	5.66	50	100

### 2.4 Quantification

Quantification of the compounds of interest was accomplished by comparison of peak areas to external standards with variations in ionization efficiency (and potential loss during ultrafiltration) adjusted using stable isotope labelled internal standards. Each class of compound, i.e. oxidized thiol, reduced thiol and sulfide-related species, has a representative internal standard. For the oxidized thiols, ^13^C_4_^15^N_2_-GSSG was used; for the reduced thiols ^13^C_2_^15^N-GSH was derivatized with an excess of NEM and used as internal standard. For sulfide, a deuterated stable-isotope labelled standard (^32^S-(d_5_-NEM)_2_) was prepared by reacting an exact amount of d_5_-NEM with a two-fold molar excess of Na_2_S in ammonium phosphate buffer pH 7.4 for 30 min at RT, and removing excess sulfide by bubbling for 60 min with high-purity argon. A mixed internal standard working solution was prepared by combining all three stable-isotope labelled compounds in ammonium phosphate buffer pH 7.4. Specific MRM conditions were established to monitor each of the internal standards (Table 1), and the ratio between the signal intensity of each analyte and its corresponding internal standard was used for quantification. Stock solutions of standards containing each analyte of interest were prepared fresh for each analysis in pH 7.4 ammonium phosphate buffer (except for cystine (CysSS) and homocysteine (HCys), which were prepared in 0.1 N HCl, while sulfide, persufide and methanethiol were first dissolved in water) and serially diluted in buffer to construct a concentration response curve for calibration; each concentration of standard was spiked with the same final concentration of stable isotope standards.

### 2.5 Derivatization of thiols and sulfide with NEM

Preliminary LC-MS/MS experiments carried out with mixed thiol standards at room temperature (RT) and pH 7.4 revealed that the reaction with NEM was complete for all analytes in under 10 min. Those results were confirmed by spectrophotometric monitoring of NEM consumption under the same conditions (302 nm; Cary 60 UV/vis spectrophotometer), demonstrating rapid kinetics for the reactions of NEM with GSH, cysteine (Cys) and Hcys (at a 1:1 molar ratio, reactions were complete within 12–15 s) whereas the reaction between NEM with sulfide (2:1 molar ratio) required 10 min to run to completion. Optimal pH was assessed by comparing reactions of NEM with either aqueous standards of select thiols (GSH, Cys, HCys, sulfide and stable-isotope labelled GSH) or by addition of NEM stocks prepared at different pH to human plasma. For those experiments, 100 mM NEM stock solutions were prepared in different ammonium phosphate buffers adjusted to pH 6.0, 7.4, 8.0 and 9.0 and used within 5 min of preparation. Freshly prepared aqueous solutions of thiols were individually reacted with NEM at these different pH values at a final concentration of 10 mM NEM, and after 5 min waiting aliquots of these NEM-thiol incubates were combined and analysed immediately. Endogenous thiols contained in human plasma (obtained by centrifugation of EDTA blood using the rapid centrifugation protocol as detailed in Section 2.8) were derivatized using freshly prepared NEM stock solutions of different pH at a 1:10 v:v ratio (10 mM NEM final concentration).

### 2.6 Assay validation

**Fig. 1:**
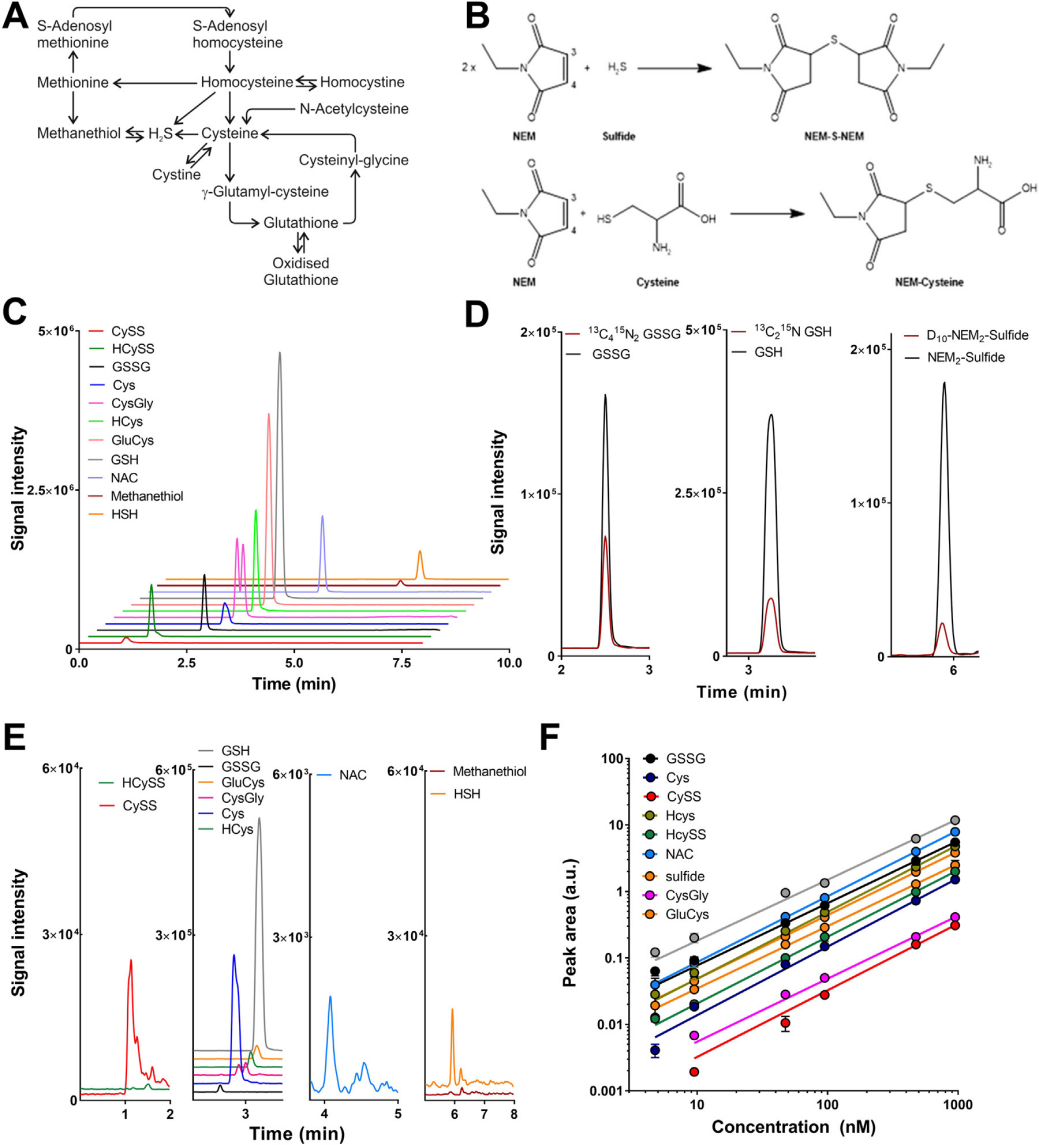
**Development and optimization of the analytical procedure for detection of the thiol redox metabolome. (A)** Metabolic pathways defining the thiol redox metabolome. **(B)** Reaction of NEM with sulfide and aminothiols (using cysteine as an example); the two positions where the sulfhydryl group can add to the double bond (Michael addition reaction with sulfur adding either to C3 or C4 of the maleimide ring) leading to the formation of two distinct diastereomers are indicated (with sulfide four different diastereomers can be formed). **(C)** Chromatographic separation and selective detection by tandem mass spectrometry of authentic stock solutions of all analytes using full registration for the entire run. **(D)** Chromatograms of stable isotope labelled internal standards. (**E)** Representative chromatogram of the same analytes at their natural abundance in human plasma using specific time windows for selected groups of compounds. **(F)** Linearity of detector response for main analytes (n = 3).

Careful assay validation for linearity, over a range of biologically relevant concentrations, and a preliminary assessment of precision, reproducibility and limits of detection were carried out using authentic standards, according to standard procedures, and are shown in Table 1 and Fig. 1. Full validation awaits further assay refinement with inclusion of additional analytes of interest.

### 2.7 Sample collection and storage

Venous blood was collected from healthy human volunteers using a 21-gauge (0.80mmx19mm) butterfly needle and BD Vacutainer™ tubes. Following gentle mixing by inversion, blood was processed immediately as described below. Saliva was collected using oral swabs (Salivette™, Salimetrics, Carlsbad, CA, USA) followed by centrifugation; a mid-stream urine sample was collected using a sterile urine container. Sample aliquots were measured either fresh or transferred into cryovials (Nunc), snap frozen in liquid nitrogen and stored at − 80 °C until analysis.

### 2.8 Sample preparation procedure and optimization

For human plasma analysis, whole blood was collected directly into EDTA BD vacutainer™ tubes and gently mixed by repeated inversion; 1 volume of NEM (100 mM stock solution in PBS) was added to 9 volumes of blood within 1 min, resulting in a final conc of 10 mM NEM (1:10 dilution), again followed by gentle mixing. Stabilized, anticoagulated blood was then centrifuged for 1 min at 3000 × *g* to separate plasma from cellular components, snap frozen in liquid nitrogen and kept at − 80 °C until analysis. Before analysis, NEM-derivatized plasma samples were supplemented with the internal standard (dual stable-isotope labelled GSSG and NEM-derivatized GSH as well as d_5_-NEM-derivatized sulfide in ammonium phosphate buffer, 1:1 v/v; target concentrations 100 nM GSH, 200 nM GSSG, 100 nM sulfide), and afterwards samples were cleared by ultrafiltration using spin columns with a 10 kD cut-off (Millipore).

Each step of the sample preparation was carefully optimized and validated. To compare detection of thiols and sulfide in plasma with that in serum, whole blood was also collected into serum BD vacutainers, incubated for 60 min at RT to allow clotting, and then centrifuged at 3000 × *g* according to the manufacturer's protocol. To test the effects of other anticoagulants, whole blood was collected into BD vacutainers containing EDTA, heparin or citrate, diluted 9:1 with NEM (final NEM concentration 10 mM), centrifuged at 800 ×*g* for 10 min at 4 °C (see Fig. 3). Centrifugation speed was optimized by comparing GSH levels in plasma obtained by standard/low-speed centrifugation of whole blood (800 × *g*, 10 min, 4 °C) with short/high-speed centrifugation (1 min 3000 ×*g* or 3 min 3000 ×*g* at 4 °C). Hemolysis was excluded by assessing plasma free hemoglobin concentration by UV–visible spectroscopy [39]. Sample stability and stabilization conditions were determined by focussing primarily on the levels of GSH, GSSG and sulfide (although other thiols were assessed in parallel) when comparing the addition of different concentrations of NEM (1, 5, 10, 20 mM final concentration) to whole blood, or when NEM addition was delayed by specified periods of time (30 s; 5, 10, 15 and 20 min). To test the effects of time of addition of NEM in whole blood, and also the effects of freeze/thawing on stabilized samples, the same blood sample was divided into identical aliquots, which all underwent the indicated procedures (addition of NEM in whole blood or plasma, repeated freeze/thawing, etc,).

### 2.9 Bound thiols, total thiols and acid-labile sulfide

To determine the concentration of *total thiols, free thiols* and *bound-thiols* (see Box 1), NEM stabilized plasma was divided into two aliquots: (a) to assess *total thiols* (= free thiols + bound thiols) one aliquot was treated with 50 mM dithiothreitol (DTT) for 30 min, which reduces LMW disulfides and mixed disulfides with proteins; (b) to determine *free thiols* one aliquot was left untreated. In both aliquots thiol concentrations were assessed following NEM derivatization by LC-MS/MS analysis, as indicated above. *Bound thiols* were calculated by subtracting *free thiols* from *total thiols*. The same procedure was carried out with the blood cell pellet (comprising red blood cells (RBCs), white blood cells, and platelets), urine and saliva samples, except that incubation time with DTT for urine and saliva was only 10 min.

Optimal reaction conditions for the reduction of disulfides by treatment with DTT were established for each biological matrix. To this end, samples were reacted with 10, 25, 50 and 100 mM DTT for 10, 20, 30 and 60 min at RT before addition of NEM; appropriate volumes of a 100 mM NEM stock solution in ammonium phosphate buffer were then added to neutralize all SH equivalents of DTT and yield an additional 10 mM excess of NEM to ensure full capture of additionally released thiols. An alternative incubation sequence was tested in which DTT was added to NEM-treated human plasma to achieve (following neutralization of the excess NEM) a similar final DTT concentration. Results obtained with either sequence were virtually identical.

To test for the presence of *acid-labile sulfide* in blood cells, urine and saliva, 50 µl of NEM-stabilized sample was placed in a septum-sealed screw-cap vial and 200 µl 0.1 N HCl was added through the septum using a gas-tight syringe. After 10 min of incubation at RT the pH was neutralized by addition of 20 µl 1 N NaOH, followed by vortexing and addition of 20 µl 100 mM NEM through the septum. Internal standards were added after a further 10 min of incubation, and samples were then subjected to ultrafiltration followed by LC-MS/MS analysis as described above. It is important to note that acid treatment will liberate sulfide from both LMW and protein-bound sulfane sulfur (and possibly other sulfur species).

### 2.10 Alternative detection of free reduced/oxidized thiols by UPLC-QTOF analysis (accuracy and method cross-validation)

25 µl of a solution consisting of 100 mM NEM with 20 mM EDTA in PBS (final [NEM] = 10 mM; final [EDTA] = 2 mM) were added to 225 µl of whole blood (9:1 (v/v) dilution). Blood cells and plasma were separated via centrifugation of whole blood at 3000 g for 3 min at 4 °C. Plasma was collected, and proteins were precipitated by mixing plasma 1:1 (v/v) with 150 µl of an ice-cold solution containing 5% SSA and 10 mM NEM in Mili-Q water. Additionally, 15 µl of the internal standard (either 2 mM glutathione ethyl ester in Milli-Q water or the same dual-labelled stable isotope GSH and GSSG standard mix as indicated above) solution was added (1:10 relative to plasma volume). The sample was mixed by vortexing, sonicated for 20 s and centrifuged at 10,000 ×*g* for 10 min at 4 °C. The supernatant was collected and the pellet extracted once more with the same volume of ice-cold 5% SSA containing NEM (10 mM) solution as before. After centrifugation at 10,000 ×*g* for 10 min at 4 °C, supernatants were combined and measured with UPLC-quadrupole time of flight (QToF) mass spectrometry using an injection volume of 10 µl.

UPLC-QToF analysis was performed on an Agilent 1290 Infinity UPLC System with a binary pump and autosampler coupled to an Agilent 6550 iFunnel QToF system. For analyte separation a Zorbax Eclipse Plus C18 RRHD 2.1 × 50 mm 1.8 µm column from Agilent was used. Mobile phase A was 0.1% formic acid in Milli-Q water, and mobile phase B was acetonitrile. Separation was accomplished using the following conditions: 0–2 min: 99% A; 2–7 min: 99 − 1% linear gradient. An isocratic flow at 1% A was then run for 5 min (7–12 min) to wash the column. After 12 min in total, the proportion of A was increased again to 99% and kept constant for 4 min. The method takes a total of 16 min at a flow rate of 0.6 ml/min. Temperature was held constant at 20 °C. Analytes were ionized in positive mode (ESI) using the following conditions: gas temperature 220 °C, drying gas 12 L/min, nebulizer: 35 psig, sheath gas temperature: 330 °C, sheath gas flow: 11 L/min, capillary voltage: 2.5 kV, nozzle voltage: 1 kV and fragmentor voltage: 30 V. Analytes were determined using extracted ion chromatograms of the total ion count.

### 2.11 Statistical analysis

Analyte levels were typically assessed in technical duplicates or triplicates for at least two independent biological samples, as indicated in figure legends. Data were analysed with GraphPad Prism 7.0c for Mac. Outlier were identified by applying the ROUT method [40]. Data were tested for normal distribution by using Shapiro-Wilk normality testing. Data are reported as mean ± SD (for technical replicates) or SEM (for biological replicates), as indicated in the figure and table legends. Group differences were tested either using 1-way or 2-way ANOVA as required by the experimental setting, followed by an appropriate multiple comparison post-hoc test (Dunnet's) or *t*-test (when just two groups were being compared) as indicated in the figure legend. Significance was assumed when p < 0.05.

## 3 Results

### 3.1 Development of suitable detection and separation conditions

Method development started by selecting key compounds of interest according to the scheme summarized in Fig. 1**A**, including HCys, homocystine (HCysSS), Cys, CysSS, GSH, GSSG, cysteinylglycine (CysGly), glutamylcysteine (GluCys), and sulfide. This was followed by establishing appropriate conditions for the detection of these analytes by LC-MS/MS. To this end, authentic standards of reduced thiols were reacted with excess NEM in buffered solution at pH 7.4 and mixed with disulfide standards to find suitable separation conditions by UPLC (see reaction scheme for reduced thiols and sulfide with NEM depicted in Fig. 1**B**). During the initial phase of development a standard C_18_ reversed phase column with a simple gradient elution profile was used for analyte separation, allowing us to make a number of general observations regarding their elution profile.

We found that symmetric and mixed disulfides (which do not react with NEM as they lack a free thiol group) show limited retention on reversed phase material, clustering together close to the solvent front; this was followed by the group of maleimide-adducts of aminothiols, with S-(NEM)_2_ eluting last. Thus, derivatizing reduced thiols with NEM clearly prolongs their retention time on the column, particularly when the derivatization product involves two succinimide groups, as with sulfide. For some compounds more than others double peaks were observed, a phenomenon previously described for Cys and GSH [41], [42]. This has been attributed to the formation of two diasteromers that differ in position of the sulfur atom in relation to the maleimide nitrogen, depending on where the reduced thiol adds to the double bond of the pyrrole ring. Modifying elution conditions afforded reasonably good separation of NEM-containing adducts, but remained problematic for disulfides. We therefore opted for a multimodal resin in order to improve retention of the highly polar analytes. The mixed mode Aqua UHPLC column was found to offer suitable properties, combining polar separation characteristics with the stability and non-polar separation of a traditional C_18_ column. This allowed for better separation of the more polar thiols such as GSSG and HCysSS; however, CysSS was still not retained well by the column and eluted with a poor peak shape close to the solvent front. The order of elution of other derivatives remained largely the same (Fig. 1**C**). Therefore, further optimization experiments were carried out on the Aqua UHPLC column according to the separation procedure described in Section 2.3 of the Materials and Methods section. Table 1 lists the structures of analytes of interest along with the specific cone/collision energies used for their detection and typical retention times observed using these separation conditions.

### 3.2 Internal standards

Since GSH is a major intracellular antioxidant and the GSH/GSSG ratio in plasma and tissues widely regarded as the ‘gold standard’ for the assessment of systemic or cellular redox status, we chose stable-isotope labelled GSH and GSSG to account for variations in ionization efficiency and possible loss of analyte during sample preparation. Consistent with the known dynamics of glutathione in blood [9] on spiking fresh or previously frozen plasma we observed a rapid loss of internal standard, unless plasma was treated with NEM before addition. Since this was observed with both reduced (fast) and oxidized (slower) internal standard, we opted to alkylate the stable-isotope labelled GSH standard and only added internal standards to the biological sample pre-incubated with NEM for at least 10 min. This measure effectively prevented any loss of internal standard even after sample deproteinization (see Section 3.9 below). The disappearance of both reduced and oxidized thiols was also observed on spiking blood plasma with Cys and HCys.

Considering the unique chemistry of sulfide and its reaction with two instead of one molecule(s) of NEM (Fig. 1**B**), we opted to include an additional stable-isotope labelled internal standard for this analyte. Preliminary studies with ^34^S-(NEM)_2_ showed that the degree of ionization quenching of the sulfide adduct in a variety of biological matrices indeed differed from that of the dual-labelled GSH adduct on occasion, justifying a separate internal standard for sulfide. However, those earlier experiments also revealed that this particular compound was not ideal as an internal standard; besides only differing by two *m/z* units (^34^S versus ^32^S) the natural abundance of the ^34^S isotope (4.2%) means that high concentrations of endogenous sulfide can lead to interference, necessitating the introduction of cumbersome mathematical correction factors. This problem was overcome by the use of the pentadeuterated NEM analogue, N-ethyl-d_5_-maleimide (d_5_-NEM) in preparing the internal standard, which coelutes with the regular NEM adduct of sulfide without showing any overlap due to the large difference of 10 *m/z* units.

Therefore, a defined mixture of ^13^C_4_^15^N_2_-GSSG, ^13^C_2_^15^N-GS-NEM and ^32^S-(d_5_-NEM)_2_ was prepared in ammonium phosphate buffer pH 7.4 and added to the mixture of internal standards of all analytes reacted with NEM, and compounds were detected using the specific MRM conditions previously established by direct infusion of individual compounds as listed in Table 1. The ratio between the signal intensity of each analyte and its corresponding internal standard was used for quantification in the biological matrix (Fig. 1**D**).

### 3.3 Linearity, precision, limits of detection and reproducibility of the method

A standard calibration set was prepared to determine linearity, precision, limits of detection (LOD), applicability and reproducibility of the method, whilst also allowing quantification of the analytes in different biological matrices.

#### 3.3.1 Linearity

Accurately weighed amounts of analytical-grade reduced thiols were dissolved in the appropriate volume of NEM-supplemented ammonium phosphate buffer and allowed to react for 5 min at a 10-fold molar excess of NEM over thiol. Accurately weighed sulfide was first dissolved in water before addition of a defined volume of this concentrated stock to NEM-containing buffer in a septum-sealed reaction vial under otherwise identical reaction conditions. NEM-derivatized thiol and sulfide solutions were then combined with roughly equimolar concentrations of the oxidized thiols to yield a stock solution of all analytes to be tested. This mixed standard was serially diluted with buffer to yield targeted final concentrations of 10 μM, 5 μM, 1 μM, 500 nM, 100 nM, 50 nM, 10 nM and 5 nM when added to the biological sample (2 × more concentrated in the mixed standard). For most compounds this is well within the physiological range, except for cellular/tissue GSH levels (being in the low mM range), which may therefore necessitate appropriate dilution of samples before analysis. To each dilution of the standard calibration set and the biological samples the same volume of internal standard was added yielding final concentrations of 100 nM GSH, 200 nM GSSG, and 100 nM sulfide. This allowed adjustment of peak areas by taking into account the ratios between analyte and internal standard. All analytes showed excellent linearity over a wide range of concentrations (> 3 orders of magnitude), with overall r^2^ values of between 0.994 and 0.999 (Fig. 1**F**).

#### 3.3.2 Sensitivity and LOD

Using an injection volume of 5 µl achieved sensitivity limits were all in the nanomolar range, differing by class of compound and structure, with a general order of disulfide>thiol adduct with one NEM>sulfide (two NEM/thiol). Limits of detection and quantification, defined as signal-to-noise ratio of > 3 and > 10, are listed in Table 1. Assay precision will be determined at a later stage of method development.

#### 3.3.3 Applicability and reproducibility

All analytes of interest could be readily detected in fresh human plasma and quantified without problems in each sample measured. Between-run reproducibility was tested for a pooled plasma sample over several days and was better than 20% overall. Fig. 1 shows a typical chromatogram of standards (**panel C**) and a representative plasma sample (**panel E**).

Our next efforts focussed on exploring what effects variations in reaction conditions such as pH and sample preparation (blood collection, cell separation, and stabilization) may have on analyte concentrations detected.

### 3.4 Optimization of reaction conditions for thiol derivatization with NEM

Reaction conditions were optimized to perform well under biologically relevant conditions. At neutral pH, NEM rapidly reacts with thiols [43], and this was experimentally confirmed to extend to physiological pH using a thiol standard mixture. Since the reaction was found to be complete in under 10 min no additional work was undertaken to further optimize reaction temperature or time as this was considered well within the timeframe required for the processing of biological samples, their placement into the sample compartment of the UPLC, and initiation of the first chromatographic run.

**Fig. 2:**
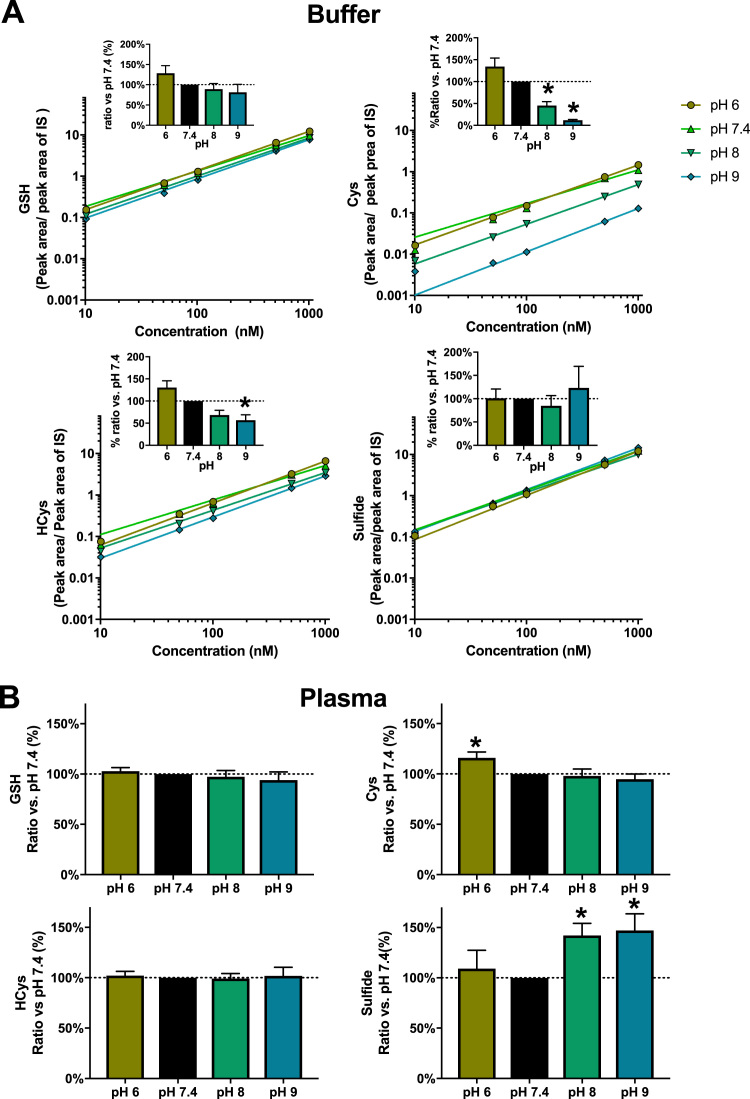
**Effects of pH on the measurement of aminothiols and sulfide using NEM.** The effect of pH was investigated in a simple aqueous buffer (A) and in plasma. (B)**. (A)** Standard curves were prepared at four different pH values: 6, 7.4 (physiological), 8 and 9, using NEM in ammonium phosphate buffer of the appropriate pH; the insets represent the percentual changes of peak area/internal standard peak area as compared to the same values obtained at pH 7.4 (black bars) for a 1 µM standard concentration. The largest impact of pH is seen for Cys with a tenfold decrease in peak areas from pH 6 to pH 9, whereas sulfide shows the least difference between the different pHs. Data are from 2 independent measurements. For GSH and sulfide the difference between groups was not significant. 1-way ANOVA p < 0.01; Dunnet's v. pH 7.4 (black bar) * p < 0.05. **(B)** NEM in ammonium phosphate buffer at each pH was added to fresh plasma samples. The percentual changes of peak area/internal standard area is presented relative to those at pH 7.4 (black bars), which was considered as a control group. Contrary to the analyses carried out in buffer (shown in panel A), the largest differences in measured peak areas were seen with sulfide, with large increases above pH 7.4 as well as a slight increase at pH 6. Data are from 6 independent samples. For GSH, and HCys the differences among the groups were not significant. 1-way-ANOVA p < 0.001; Dunnet's v. pH 7.4 (black bar) * p < 0.05.

Reaction pH is known to be critical for thiol alkylation by NEM for a number of reasons (see Discussion for details), and pH values in the range of 6.5–7.5 have been used in most previous studies. Human blood plasma deviates very little from its normal physiological pH of 7.40 under most conditions and is naturally buffered to maintain its pH. We therefore sought to experimentally test these assumptions by carrying out a series of experiments with select analytes (GSH, Cys, HCys and sulfide), varying the pH from 6 to 9 in an aqueous buffer system; those experiment were complemented by additional incubations of NEM with human plasma at the same pH values. The results of these studies are displayed in Fig. 2.

The results reveal clear differences between the reaction of NEM with structurally distinct thiols. Whereas little difference was found for the calibration curves for sulfide between pH 6 and 9, small differences were observed for GSH; these differences became increasingly prominent with HCys and Cys. With Cys, a 10-fold difference in product yield was observed between pH 7.4 and pH 9. When fresh human plasma was added to NEM dissolved in buffer of different pH, the changes in peak areas were not as dramatic as with the mixture of reduced thiols in aqueous solution. Yet, a few notable differences were observed: peak areas decreased slightly with alkalinization for GSH and Cys and increased rather more markedly for sulfide; a small increase compare to physiological pH was also observed upon acidification with Cys and sulfide.

Taken together, this data suggests that adjustment of the reaction pH from 7.4 to either more acidic or more alkaline conditions affects product yield and/or stability of the reaction product to a different extent with different thiols, with the most dramatic variation observed with Cys. If the calibration curves of the authentic standards at the respective pH would be used, this would result in an overestimation by a factor of 10 in the case of Cys. For sulfide, the same changes in pH appear to result in increased sulfide amounts to become available for reaction with NEM; whether this was a result of decomposition of metastable per/polysulfides or due to enhanced release of sulfide from non-covalent binding sites remains unclear at this stage. All further experiments were therefore carried out using NEM-containing stock solutions kept at pH 7.4. Since the physiological pH of plasma, saliva and cellular samples is almost always near this pH there is in fact no need to add a stabilizing buffer for these compartments; however, the pH of urine may vary from slightly acidic to alkaline, depending on nutritional habits and metabolic status; thus, checking pH after addition of NEM-containing buffer would seem to be advisable.

### 3.5 Versatility for assesement of other thiols and sulfur metabolites

Having demonstrated that in principle our platform technology is robust and allows sensitive, specific and simultaneous measurement of multiple thiols, disulfides and sulfide in plasma our next efforts focussed on demonstrating its versatility. To this end, we tested how cumbersome it would be to include an additional analyte into the existing method. Herein we show examples for three very different compounds: methanethiol (methyl mercaptan, CH_3_SH), N-acetylcysteine (NAC), and coenzyme A (CoA). Establishing specific MRMs, optimizing cone/collision energies and subsequently testing where the compound elutes on the column takes approximately one day per analyte using UPLC-MS/MS. NAC and CoA did not differ much from other thiols in terms of fragmentation properties. CH_3_SH was found to be extremely sensitive to in-source fragmentation, requiring a very low cone voltage for detection; it was also very well retained on the column, eluting between the other thiols and sulfide (see Fig. 1**C**). NAC fell within the same time window where other aminothiols elute, whereas CoA showed an unusually strong interaction with the mixed mode column. This resulted in extreme peak tailing, preventing inclusion of CoA in the current method. Ongoing efforts focus on developing a short method for discrete measurement of this particular analyte using a C_18_ column. Thus, in principle, many new analytes can be incorporated into the method within one day, although some compounds (here exemplified by CoA) may require additional development effort and/or alternative chromatography/elution conditions.

### 3.6 Optimization of sample preparation procedures for plasma: protein removal and anticoagulant

To allow this method to be used in translational studies [3], we focussed on the optimization of analysis of thiols and sulfide primarly in biological matrices. Since blood is in contact with all tissues, it is likely to represent not only a convenient biomarker matrix but also to play a central role in systemic thiol and sulfide metabolism. A further advantage is that blood is relatively easily accessible. Therefore, in the present study we focused on optimizing sample preparation for blood (although these procedures can be easily adapted to tissues and cells).

To avoid changes in thiol speciation (in particular sulfide) while minimizing interference with chromatographic separation and analyte detection, we opted to remove proteins by simple ultrafiltration over a 10 kDa membrane. The advantages of this approach to sample clean-up over protein precipitation methods include the avoidance of analyte dilution, pH changes and addition of organic solvents, which may interfere with the separation (please see Discussion for further comments).

**Fig. 3:**
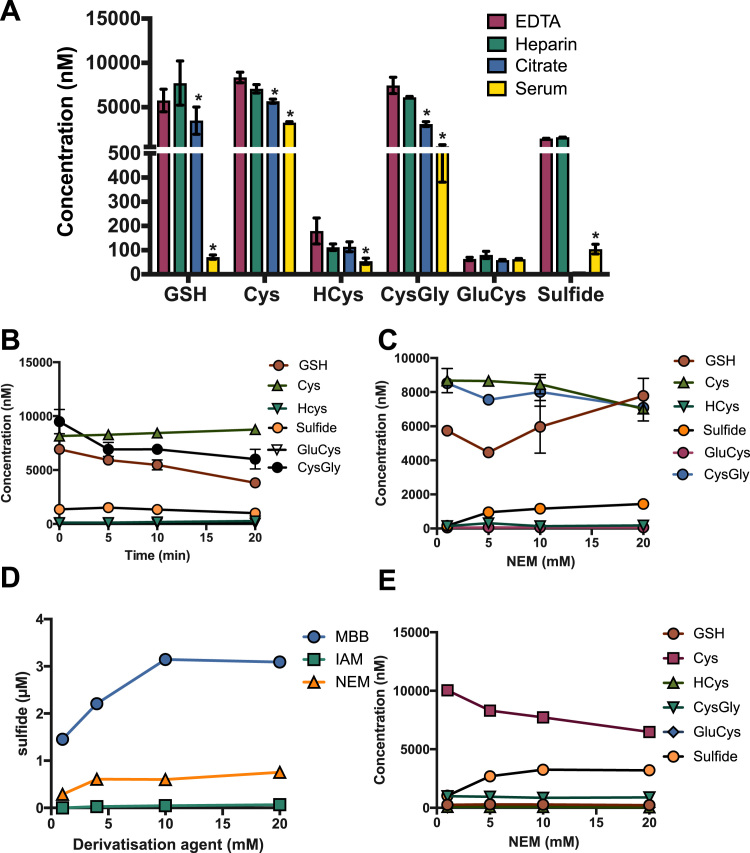
**Optimization of sample preparation procedure: anticoagulation, and stabilization with NEM.** For all experiments depicted, NEM (or other thiol alkylating agent) was added directly to whole blood (panels A-D) after which the sample was centrifuged at 800 ×*g* for 10 min at room temperature. In panel E NEM was added to plasma directly after separation from blood cells by centrifugation of whole blood. **(A)** The choice of anticoagulant affects the concentrations of thiol measured; because of its metal chelating properties EDTA is the most suitable anticoagulant for assessment of the thiol redox metabolome. 2-way ANOVA p < 0.0019, Dunnet's vs. EDTA * p < 0.01 **(B)** Delay in addition of NEM to whole blood leads to progressive decreases in GSH and CysGly concentrations as compared to time = 0. **(C)** The apparent concentrations of GSH and sulfide increase with increasing concentrations of NEM added to whole blood, as compared to whole blood treated with 1 mM NEM as a control (centrifugation 800 ×*g* x 10 min). **(D)** The concentration of sulfide detected increases with increasing concentrations of alkylating agents added to whole blood**. (E)** The NEM concentration-dependent increases of GSH levels (as compared to samples treated with 1 mM NEM) were not observed when NEM was added to plasma (although also absolute GSH concentrations were considerably lower), suggesting that these increases were largely due to leakage of GSH from blood cells; however, apparent plasma sulfide concentrations still increased with increasing NEM concentrations, suggesting removal of sulfide from bound forms in plasma at elevated concentrations of the alkylating agent. For all panels data were obtained by analysis of two independent biological samples taken form different human individuals (mean ± SD); measurements were carried out at least in duplicate.

The choice of anticoagulant used to collect blood may have a significant impact on the concentrations of thiols/disulfides and sulfide measured, and previous work on glutathione emphasized the importance of metal chelators to minimize artificial oxidation [44]. Our first efforts to identify optimal conditions for combined thiol/sulfide analysis in whole blood therefore focussed on the choice of anticoagulant (Fig. 3**A**). For reasons of practicality we limited our comparison to using commercial vacutainer tubes. In all cases, NEM was added immediately after filling and before centrifugation at 800 ×*g* for 10 min (the standard protocol used in many of our earlier studies). A final sample concentration of 10 mM NEM was chosen in all the pilot work. This had been successfully used by our group and others in the context of NO related research to prevent changes in concentrations of nitrite, nitrate and nitroso species in blood and tissues [45].

Blood was collected from three different individuals into EDTA, heparin, citrate or serum vacutainers and tested in direct comparison (Fig. 3**A**). Citrated samples had a greatly reduced sulfide concentration compared to other anticoagulants, likely due to the acidic nature of this anticoagulant perhaps favoring volatilization of sulfide. Blood collected in heparin tubes showed slightly higher levels of GSH and sulfide compared to EDTA, but lower levels of Cys and CysGly. Serum tubes were unsatisfactory for measurement of physiological levels of thiols and sulfide as they showed greatly reduced concentrations of the majority of thiols measured (except GluCys) and sulfide. Whether this was due to excessive protein thiolation as a result of the coagulation process remains speculative at this juncture and was not further investigated. In any case, these results seem clearly to rule out serum or citrate tubes for thiol redox measurements. Since the concentrations of most analytes measured were rather similar using either EDTA or heparin anticoagulated blood and the former may have an advantage inasmuch as it minimizes thiol oxidation by metal chelation (obviating the need to add another chelating agent) all further validation experiments were carried out using EDTA as anticoagulant.

### 3.7 Sample stabilization with NEM

All thiols are susceptible to oxidation when exposed to ambient air, and sulfide is in addition volatile; it would thus seem advisable to add alkylating agents such as NEM as soon as possible to a biological sample in order to avoid artificial shifts in the ratio of reduced to oxidized thiols. For blood, we reasoned that it would be advantageous to add NEM immediately after collection. As expected, delaying the addition of NEM to whole blood resulted in lower levels of GSH being detected in plasma (Fig. 3**B**). In contrast, sulfide showed an apparent increase in concentration the longer the blood was left without derivatization. Irrespective of specific mechanisms involved it would seem advisable to add the alkylating agent as soon as feasible to the biological sample of interest.

Our next efforts revolved around finding the optimal concentration of NEM to achieve complete alkylation (Fig. 3**C**). The most abundant thiol in human plasma is the single free sulfhydryl group of Cys-34 of serum albumin [46], which is not analysed in this method but will still react with (and thus consume) NEM. It has a plasma concentration in the order of 0.3–0.4 mM in healthy human individuals; to some extent, NEM can also react with amine residues consuming additional equivalents. Thus, to ensure that all of the low-molecular weight thiols and sulfide are fully derivatized a range of NEM concentrations (1–20 mM final concentrations) were tested. There was no significant change in concentration between 1 mM and 5 mM NEM for the majority of analytes. However, the concentration of NEM used for derivatisation of whole blood had a dramatic effect on the apparent plasma levels of GSH and sulfide recorded (Fig. 3**C**). Higher NEM concentrations significantly increased the levels of GSH and sulfide measured, while those of other thiols were either not affected or their concentrations were slightly reduced. Sulfide levels showed the largest variation with different NEM concentrations, tripling between 1 mM and 10 mM NEM and plateauing between 10 mM and 20 mM (Fig. 3**C**).

A striking observation was that, at the two highest concentrations of NEM tested (10 and 20 mM), measured plasma GSH concentration was considerably higher than the range typically reported in the literature [44]. By contrast, GSH and sulfide concentrations increased to a much lesser extent when NEM was added to plasma after its separation from RBCs (and other blood cells) by centrifugation (compare Fig. 3**E** and **C)**. This data suggests that the observed elevated plasma concentrations of these two analytes, when higher NEM concentrations were added to whole blood, were an artefact resulting from leakage and/or transport of material from blood cells. This phenomenon was not specific to this particular thiol alkylating agent since even higher levels of sulfide were found when mBB instead of NEM was used as thiol alkylating agent and added to whole blood under otherwise identical conditions. A direct comparison of mBB, NEM and IAM (Fig. 3**D**) revealed that the latter resulted in the lowest sulfide levels detected, demonstrating that the nature of the alkylating agent applied can have a marked influence on the levels of sulfide detected.

**Fig. 4:**
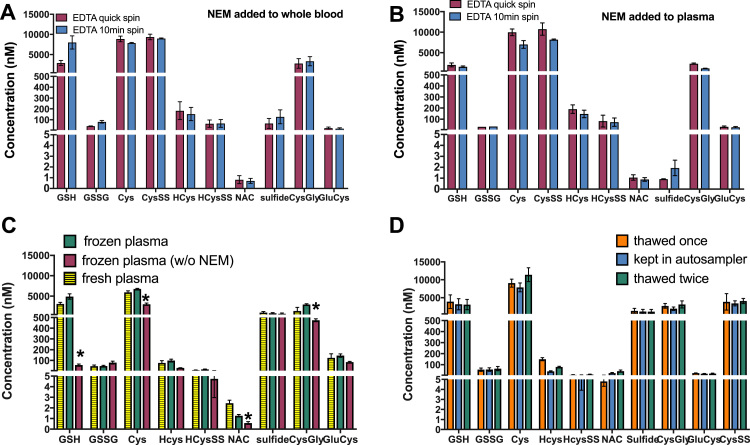
**Optimization of sample preparation procedure: effects of centrifugation speed and duration, effects of freeze/thawing and sample stability. (A,B)** NEM addition to whole blood (shown in A) increases stability of thiols in comparison to NEM addition to plasma (shown in B), as demonstrated by comparing overall absolute concentrations of the same thiols in A vs. B. However, when low speed/long duration centrifugation (800 ×*g*, 10 min) was chosen to separate plasma form blood cells this procedure artificially increases plasma GSH concentrations (shown in A, pink bar vs. blue bar), probably due to leakage of NEM-adducts form cellular blood components (RBCs); see main text. **(C)** Freezing of NEM stabilized plasma leads to an increase in GSH, Cys and CysGly concentrations compared to their levels in fresh samples; concentrations of GSH dramatically decreased, and GSSG increased when samples were frozen without stabilization by NEM (n = 3; 2-way RM ANOVA p < 0.001; Dunnet's vs. fresh plasma p < 0.01). **(D)** In samples stabilized with NEM freeze/thawing or maintenance of samples in thermostatted autosampler at 5 °C did not affect concentrations of the redox thiol metabolome (n = 3, differences among groups are not significant). For all panels 2 or 3 independent biological samples from different individuals were analyzed; measurements were carried out in triplicates. (For interpretation of the references to color in this figure legend, the reader is referred to the web version of this article).

As centrifugation time itself contributes to the delay between collection of blood and prevention of thiol oxidation by addition of NEM and consequent loss of GSH (as observed in Fig. 3**B**) and other thiols the centrifugation protocol was also optimized for centrifugation speed (800–3000 ×*g*), centrifugation time (1–10 min) and hemolysis of RBCs (Fig. 4**A,B**). The latter is of critical importance due to the very high concentration of GSH and sulfide within RBCs, such that small numbers of ruptured cells can lead to dramatic increases in the apparent plasma concentration of these analytes. None of the centrifugation protocols tested induced hemolysis of RBCs as assesed by UV–vis spectrophotometry of the plasma obtained after centrifugation, a simple procedure that can detect concentrations of free hemoglobin as low as 10 nM (a low-grade hemolysis corresponds to absorbance values of > 0.2 [39]). Nevertheless, a consistent finding was that the longer/low-speed centrifugation (800 ×*g*, 10 min) resulted in considerable leakage of GSH from the cells (Fig. 4**A – GSH**, compare pink vs. blue bar).

Therefore, the optimal protocol chosen to preserve the natural speciation of thiols and sulfide in blood was to add NEM to a final concentration of 10 mM as quickly as possible upon collection of blood, combined with a short/rapid centrifugation procedure (1 min at 3000 ×*g*).

### 3.8 Sample storage and stability

For the most part, samples will have to be collected over longer periods of time (and in case of multicenter studies even at different locations) requiring storage in the frozen state before analysis. We therefore felt it was important to ensure that signal intensities of key analytes did not suffer a dramatic loss upon freeze/thaw. As shown in Fig. 4**C**, instead of any significant decrease, some concentrations notably increased after freeze/thawing, especially CysGly whereas others such as sulfide did not change at all. The only exception was NAC, which dropped by 30–50% after a single freeze/thaw compared to immediate analysis. However, another obvious finding from these studies was that the extent of concentration changes observed varied dramatically from individual to individual and between analytes, ranging from a few percent to > 3-fold. Further stability tests with NEM-treated samples showed no significant changes in the levels of the analytes when kept for extended time in the refrigerated autosampler compartment or subjected to an additional freeze/thaw (Fig. 4**D**). Therefore, human plasma samples stabilized with NEM can be frozen without apparent loss in concentration of analytes; however, absolute concentrations measured differ from those apparent on immediate sample analysis.

### 3.9 Accuracy for measurement of glutathione redox status and comparison of analytical results

**Fig. 5:**
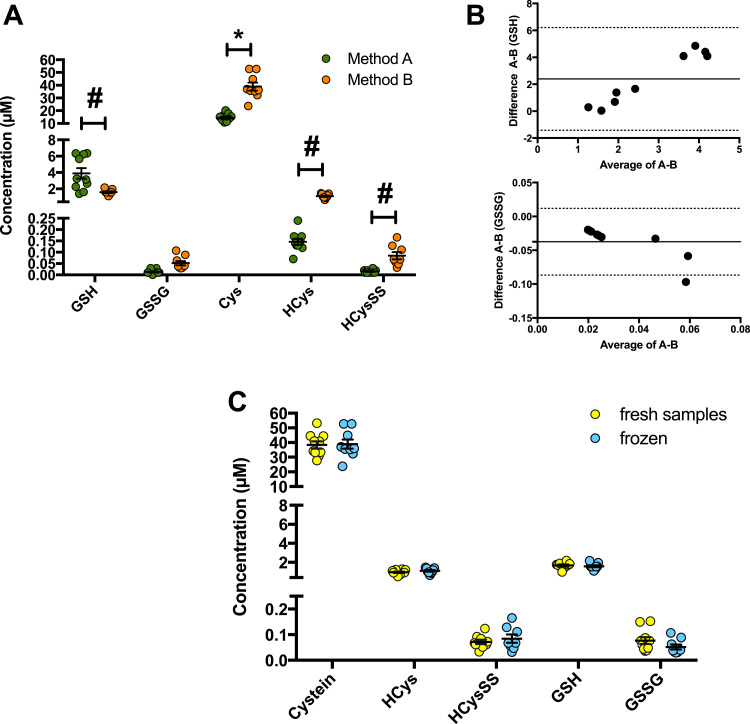
**Determination of the accuracy of the method (Method A) by comparison with an independent method optimized for GSH/GSSG detection (Method B) in plasma samples from 10 healthy human volunteers**. In Method A detection of the thiol redox metabolome was carried out after ultrafiltration, separation on the Aqua UPLC column and detection by triple-quadrupole mass spectrometry. In Method B, thiols and disulfides were measured following deproteinization with sulfosalicylic acid, separation on a C_18_-UPLC column, and detection by Q-ToF mass spectrometry. **(A)** Concentrations of GSH are grossly underestimated by Method B, while Cys, HCys and HCysSS are overestimated by Method B compared to Method A (n = 10; 2-way RM ANOVA Method A vs. B p < 0.0001; Sidak's multiple comparison test p < 0.01; # T-Test-p < 0.01). **(B)** Bland-Altman plots of the absolute differences in GSH and GSSG concentrations as assessed by Method A - Method B in relation to the mean values of the analytes. **(C)** Effects of freeze/thawing were absent when Method B (with SSA deproteinization) was used (n = 3). Both methods revealed very similar values when the same sample clearing/deproteinization procedures were applied. For all panels 3–10 independent biological samples were analysed; measurements were carried out in triplicates.

We sought to demonstrate the accuracy of our platform technique by comparing the results obtained by an independent laboratory. Instead of ultrafiltration, this laboratory used a protein removal technique accepted and optimized for GSH/GSSG mesurements, i.e. by precipitation with sulfosalicylic acid (SSA), along with a different chromatographic method (regular C_18_ instead of mixed mode material) and mass spectrometry principle (ToF high resolution instead of triple quadrupole; see Methods for details). Two other thiols (Cys, HCys) and one disulfide (HCysSS) were also included in the comparison in addition to GSH/GSSG. The concentrations of these analytes were compared between both methods in plasma from 10 healthy human volunteers (Fig. 5). On comparison of Method A (ultrafiltration and triple quadrupole) with Method B (SSA deproteinisation and QToF) we found that deproteinization of samples with SSA leads to a statistically significant underestimation of GSH and GSSG, while Cys, HCys, HCySS yielded consistently higher concentrations (Fig. 5**A**). These differences are evident on Bland-Altman comparison of the methods (Fig. 5**B**).

### 3.10 Assessment of total and bound thiols by treatment with DTT

**Fig. 6:**
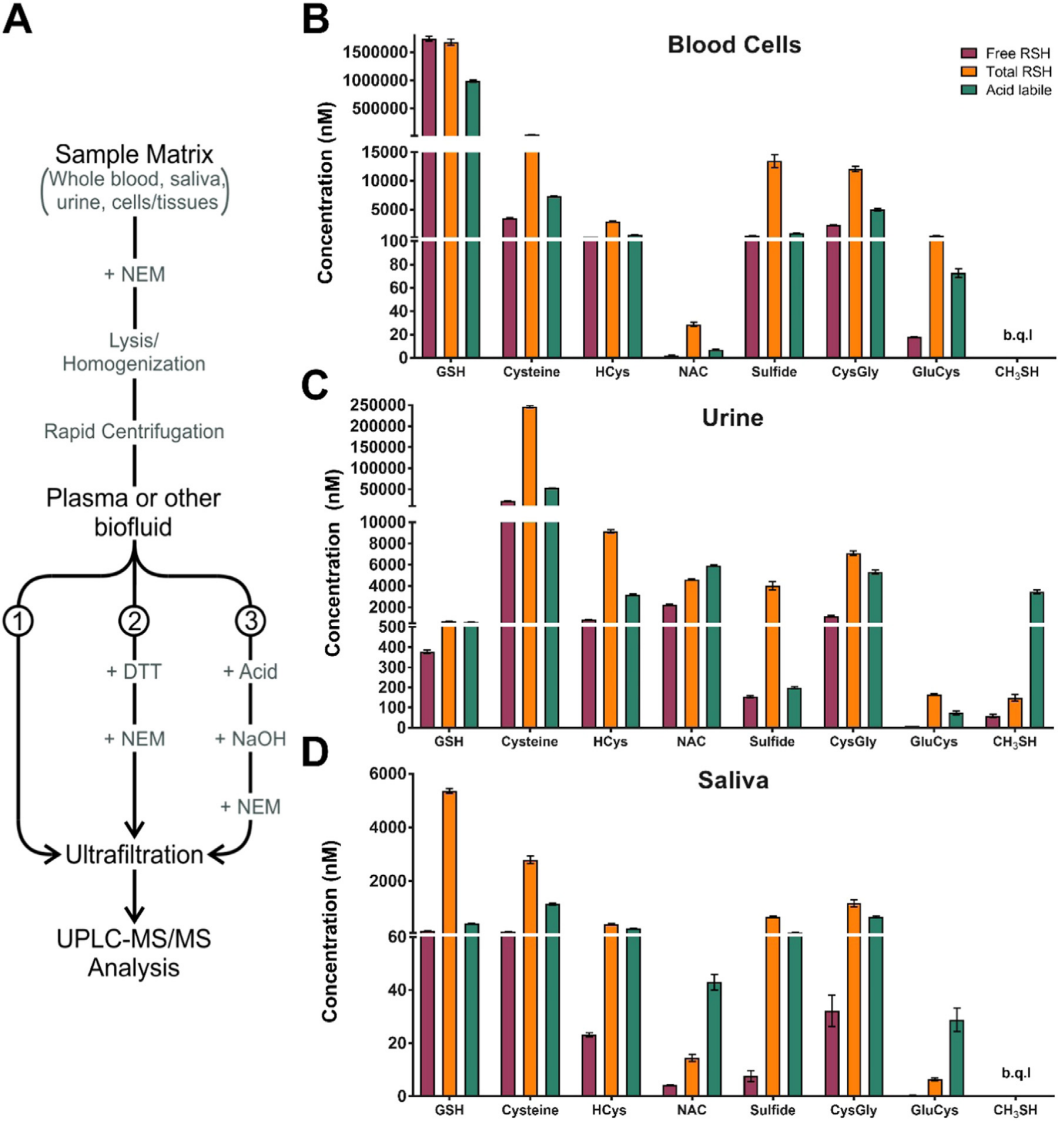
**Assessement of*****free*****,*****total*****and*****acid-labile*****thiols in human blood cells, urine and saliva.** (**A**) Workflow for determination of free (1), total (2) and acid-labile thiols (3) in biological samples such as blood plasma (Table 2), blood cells (panel B) and other biofluids (panels C,D). (**B-D**) For the determination of *free*, *total* and *acid-labile thiols* in blood cells (depicted in **B**), urine (shown in **C**) and saliva (shown in **D**), samples were divided into 3 aliquots; one aliquot was used for determination of *free thiols*, one for *total thiols* following DTT reduction, and one for *acid-labile thiols* after addition of hydrochloric acid (HCl) and subsequent neutralization by sodium hydroxide (NaOH). All measurements were carried out in triplicate. Data in **B** are means from individually analysed cell pellets of the same 10 volunteers of whom circulating plasma thiol concentrations were analysed for inclusion in Table 2; data in panels **C** and **D** were from pooled saliva and urine samples of 5 healthy volunteers.

*Total* amounts of a specific *thiol* in a biological sample can be determined by reduction of all disulfides by DTT before NEM addition and analysis, while the corresponding *free thiol* is assesed in DTT untreated samples. The *bound thiol* levels are obtained by calculating the difference of *total thiols*-*free thiols*. First, to ensure complete reduction of all disulfides (including sulfide) we determined the minimally effective concentration of DTT and the incubation time required to achieve the highest final concentration of LMW thiols and sulfide in the reaction solution. Complete reduction was achieved by treatment of plasma with 50 mM DTT for 10 min at RT (sample workflow is shown in Fig. 6**A**). Similar results were obtained by analyzing other biofluids, including saliva and urine, whereas human blood cells required 100 mM DTT and 30 min incubation for complete reduction (see below).

There were no significant differences in the final concentration of thiols in plasma samples reacted with NEM before reduction compared with samples reduced with excess DTT and then reacted with NEM.

As a proof-of-concept study, we determined the steady-state concentrations of *free reduced and oxidized thiols* as well as the *total thiol* content in plasma of 10 healthy human volunteers (Table 2). The concentrations listed correspond broadly to values previously reported in the literature [46]. However, the distribution of circulating concentrations among healthy human volunteers highlights a number of aspects that are usually less obvious by looking at averages. First, there are considerable differences in concentration of free thiols between individuals, varying from 2 to 3 fold in some cases to as much as 10-fold for other analytes. Second, the ratios of reduced over oxidized thiols varies markedly between glutathione, cysteine and homocysteine. Third, amounts of bound thiols are generally higher than circulating concentrations of free reduced thiols; this is particularly striking for GluCys and sulfide for example, where bound sulfide exceeds free sulfide by a factor of 100. Persulfide and methanethiol were not detected in any plasma sample. However, solutions of NEM-derivatized standards of persulfide (formally, the disulfide of H_2_S in its anionic form) were found not to be stable over time, representing a methodological limitation that is not easily overcome without switching to another alkylating agent.

**Table 2:** Free, total and bound concentrations of the 10 aminothiols and sulfide measured in human plasma from 10 individuals and redox ratios of the key redox pairs. *The measured concentrations of CySS are not physiologically accurate due to poor peak shape and ion suppression. Columns 1–10 show the mean ± SD of 3 technical replicate measures performed on plasma from 10 individuals. Outliers were identified by applying the ROUT test, marked in italics, and excluded from descriptive statistics (mean±SEM).

		**1**	**2**	**3**	**4**	**5**	**6**	**7**	**8**	**9**	**10**	**Mean ± SEM**
**GSH**	Free / μM	6.34 ± 0.04	2.65 ± 0.05	1.40 ± 0.02	3.24 ± 0.01	6.36 ± 0.11	1.60 ± 0.04	2.26 ± 0.04	3.14 ± 0.08	6.25 ± 0.23	5.67 ± 0.18	3.89 ± 0.65
	Total / μM	9.56 ±0.69	7.33 ± 0.94	5.48 ± 0.22	9.49 ± 0.32	9.26 ± 0.88	5.69 ± 0.38	5.78 ± 0.24	6.75 ± 0.47	8.42 ± 0.37	8.35 ± 0.45	7.61 ± 0.51
	Bound / μM	3.22 ± 0.66	4.68 ± 0.97	4.07 ± 0.21	6.24 ± 0.32	2.90 ± 0.98	4.09 ± 0.35	3.52 ± 0.23	3.61 ± 0.39	2.17 ± 0.18	2.69 ± 0.48	3.72 ± 0.36
											
**GSSG**	Free / nM	7.17 ±0.34	*31.86 ± 2.55*	6.01 ± 0.40	9.76 ± 0.91	6.24 ± 0.89	*26.41 ± 2.24*	6.86 ± 0.81	5.00 ± 0.57	7.89 ± 0.53	7.80 ± 0.59	7.09± 0.51
												
**GSH/GSSG**	Redox Ratio	884	83	234	332	1020	61	329	628	792	726	509 ± 109
												
**Cys**	Free / μM	15.98 ± 0.54	22.3 ± 1.46	10.81 ± 0.47	17.72 ± 0.46	15.14 ± 1.15	12.65 ± 0.71	15.42 ± 0.62	13.50 ± 0.44	9.81 ± 0.34	11.28 ± 0.28	14.46 ± 1.18
	Total / μM	210.01 ± 26.96	198.81 ± 13.25	188.68 ± 1.82	177.75 ± 5.52	195.03 ± 4.37	242.2 ± 8.76	155.08 ± 0.48	215.78 ± 11.31	125.86 ± 12.10	168.66 ± 10.73	187.8 ± 10.43
	Bound / μM	194.03 ± 27.48	178.59 ± 22.71	177.87 ± 1.66	160.02 ± 5.46	179.9 ± 5.45	229.55 ± 8.20	139.66 ± 0.79	202.28 ± 10.88	116.04 ± 11.83	157.39 ± 11.00	173.5 ± 32.26
											
CySS*	Free / μM	*14.90 ± 0.69	*15.10 ± 0.34	*11.42 ± 0.05	*12.45 ± 0.43	*13.22 ± 0.32	*19.96 ± 5.28	*9.06 ± 0.27	*14.78 ± 0.63	*10.59 ± 0.69	*12.22 ± 0.49	*13.37 ± 0.96
												
Cys/CySS*	Redox Ratio	*1.07	*1.48	*0.95	*1.42	*1.15	*0.63	*1.70	*0.91	*0.93	*0.92	*1.12 ± 0.10
												
**Hcys**	Free / μM	0.15 ± 0.002	0.24 ± 0.004	0.13 ± 0.005	0.17 ± 0.005	0.17 ± 0.005	0.14 ± 0.006	0.14 ± 0.003	0.07 ± 0.001	0.12 ± 0.004	0.13 ± 0.002	0.15 ± 0.01
	Total / μM	8.62 ± 0.49	5.47 ± 0.46	5.21 ± 0.17	7.81 ± 0.21	5.65 ± 0.21	9.59 ± 0.33	6.02 ± 0.42	6.37 ± 0.09	4.40 ± 0.57	7.95 ± 1.31	6.71 ± 0.53
	Bound / μM	8.47 ± 0.49	5.23 ± 0.45	5.08 ± 0.16	7.64 ± 0.21	5.48 ± 0.21	9.45 ± 0.34	5.88 ± 0.42	6.29 ± 0.09	4.29 ± 0.57	7.82 ± 1.31	6.56 ± 0.53
											
**HcySS**	Free / nM	18.95 ± 0.78	15.92 ± 1.44	9.72 ± 3.54	22.08 ± 1.16	11.39 ± 0.74	29.08 ± 11.08	11.7 ± 1.39	5.94 ± 2.32	13.00 ± 2.01	14.22 ± 1.15	15.2 ± 2.12
												
**Hcys/HcySS**	Redox Ratio	8.05	15.36	13.06	7.54	14.84	4.64	11.76	12.15	8.95	8.86	10.52 ± 1.09
												
**Sulfide**	Free / μM	0.27 ± 0.020	0.29 ± 0.016	0.03 ± 0.001	0.28 ± 0.005	0.05 ± 0.001	0.22 ± 0.017	0.30 ± 0.013	0.06 ± 0.010	0.11 ± 0.011	0.04 ± 0.001	0.17 ± 0.04
	Total / μM	15.82 ± 3.78	15.83 ± 3.34	16.50 ± 2.92	15.45 ± 0.98	12.66 ± 3.73	16.09 ± 1.75	17.82 ± 0.60	16.35 ± 2.30	17.90 ± 1.85	19.55 ± 3.12	16.4 ± 0.58
	Bound / μM	15.55 ± 3.77	15.54 ± 3.35	16.46 ± 2.92	15.16 ± 0.99	12.60 ± 3.73	15.87 ± 1.76	17.52 ± 0.60	16.29 ± 2.29	17.79 ± 1.84	19.51 ± 3.12	16.23 ± 0.58
											
**Cys-gly**	Free / μM	1.47 ± 0.08	1.74 ± 0.10	1.12 ± 0.01	1.63 ± 0.06	1.91 ± 0.05	1.65 ± 0.05	1.41 ± 0.02	1.35 ± 0.02	1.64 ± 0.01	1.02 ± 0.05	1.49 ± 0.09
	Total / μM	27.38 ± 0.96	29.75 ± 0.42	24.28 ± 0.37	28.66 ± 0.48	34.13 ± 1.08	41.15 ± 1.18	28.31 ± 1.20	29.11 ± 0.18	19.61 ± 0.54	26.31 ± 0.60	28.87 ± 1.81
	Bound / μM	25.91 ± 0.94	28.01 ± 0.48	23.16 ± 0.37	27.03 ± 0.51	32.22 ± 1.04	39.50 ± 1.22	26.90 ± 1.18	27.77 ± 0.20	17.97 ± 0.53	25.29 ± 0.55	27.38 ± 1.78
											
**Glu-cys**	Free / μM	0.07 ± 0.001	0.09 ± 0.003	0.05 ± 0.001	0.09 ± 0.001	0.08 ± 0.002	0.08 ± 0.003	0.07 ± 0.004	0.07 ± 0.001	0.05 ± 0.002	0.08 ± 0.002	0.08 ± 0.004
	Total / μM	3.30 ± 0.21	2.62 ± 0.32	2.01 ± 0.41	3.02 ± 0.60	3.17 ± 0.82	3.18 ± 0.10	2.52 ± 0.11	2.34 ± 0.57	1.33 ± 0.23	2.69 ± 0.29	2.62 ± 0.19
	Bound / μM	3.23 ± 0.21	2.53 ± 0.33	1.96 ± 0.41	2.93 ± 0.61	3.10 ± 0.82	3.10 ± 0.10	2.46 ± 0.12	2.27 ± 0.57	1.28 ± 0.23	2.61 ± 0.29	2.55 ± 0.19
											
**NAC**	Free / nM	4.23 ± 0.30	5.48 ± 0.87	5.23 ± 0.15	4.40 ± 0.32	8.50 ± 0.12	4.69 ± 0.38	4.47 ± 0.18	5.12 ± 0.89	9.1 ± 0.07	*95.10 ± 2.42*	5.69 ± 0.61
	Total / nM	*428.94 ± 10.48*	35.04 ± 1.47	34.82 ± 0.79	36.32 ± 1.37	31.34 ± 1.22	34.71 ± 1.79	36.49 ± 2.08	42.77 ± 1.82	36.6 ± 2.69	*512.7 ± 5.27*	36.01 ± 1.14
	Bound / nM	*424.71 ± 10.77*	29.56 ± 0.70	29.59 ± 0.91	31.92 ± 1.07	22.85 ± 1.12	30.02 ± 2.06	32.02 ± 1.93	37.65 ± 2.21	27.51 ± 2.67	*417.6 ± 6.67*	30.14 ± 1.49

Overall, these data show that our method can also be applied for high-throughput determination of free and protein-bound thiols in human plasma.

### 3.11 Acid-labile sulfur and redox measurements in blood cells and other biofluids

In addition to the possibility of measuring free thiols and subjecting aliquots of the same biological specimen to additional reduction with excess DTT (before or even after addition of NEM) to liberate bound thiols, another sample aliquot can be pretreated with acid, neutralized and then reacted with NEM to measure *acid-labile sulfur* (and other thiol-containing species that may be released upon this treatment; see Fig. 6**A** for workflow). Fig. 6**B-D** shows exemplary results using this sample workflow for blood cells and two other biofluids, urine and saliva. It is immediately apparent from this comparison that not only absolute concentrations differ dramatically between different biospecimen, but also the distribution of thiols (i.e. the proportion of *free* versus *bound*, and *acid-labile*). Similar to plasma (Table 2), bound cysteine exceeded free levels by more than an order of magnitude in urine and saliva; however, bound glutathione was only found in saliva, not urine or blood cells. Somewhat lower levels of cysteinylation but essentially no glutathionylation were found in blood cells. Significant amounts of *acid-labile* sulfur are present in saliva, urine and blood cells. Unexpectedly, not only sulfide concentrations were increased following sample acidification, but some thiols also showed clearly higher levels compared to untreated controls. This was particularly evident for methanethiol, concentrations of which increased approximately 50-fold upon acidification in urine; methanethiol concentrations in blood cells and saliva remained below the quantifiable limit. It is important to point out that under acidic conditions the NEM-sulfide adduct is likely to originate not only from reaction with free HS^-^ but also with both protein-bound and LMW polysulfides due to the susceptibility of these compounds to acid hydrolysis.

In summary, an additional straightforward sample pre-treatment allows further expansion of the measurement opportunities to include acid-labile sulfur species in biological samples. While there seems to be some overlap between DTT-reducible and acid-labile material distinct differences remain, suggesting different chemical characteristics of the biological material susceptible to reduction and acid-treatment.

## 4 Discussion

We here outline a novel specific, highly sensitive and robust mass spectrometry-based approach for the measurement of the thiol redox metabolome capable of quantifying *total* and *free thiols*, and their corresponding *disulfides* as well and *sulfide* in complex biological matrices such as blood, saliva and urine. Specifically, we describe our validation work focusing on i) the development of the analytical procedure, i.e. suitable detection and separation conditions, quantification strategy (external and internal standards, linearity, and versatility of the method); ii) optimization of sample preparation, using blood as starting material (choice of anticoagulant, sample stabilization, centrifugation and storage); iii) versatility of the method with examples of how further compounds of interest can be included for a more comprehensive coverage of the thiol redox metabolome in the future; iv) determination of the accuracy for detection of glutathione redox status as compared to an optimized, well established method; v) analysis of the thiol metabolome (including total and free thiols and their corresponding disulfides, as well as hydrogen sulfide) in plasma samples of 10 healthy human volunteers, as well as in blood cells, urine and saliva. Taken together, this data indicates that the approach can be used to characterize the redox metabolome in patient cohorts and animal models of disease. It can therefore be applied in clinical and translational studies in search for novel prognostic and diagnostic strategies for patient stratification; moreover, it may assist in identifying novel interventional approaches for the treatment of redox diseases [3].

### 4.1 Is there a need for yet another method to determine thiol redox status?

There is no shortage of analytical methods for the determination of biological thiols (for recent reviews see [47], [48]), and many more assays appeared in just the last couple of years. Several of those more recent additions to our analytical armamentarium use a combination of some form of chromatographic separation (HPLC, UPLC or capillary electrophoresis) and mass-spectrometry for detection. In most (albeit not all) cases, the latter offers better selectivity and higher sensitivity compared to earlier spectrophotometric, fluorimetric, chemiluminescence or electrochemical detection approaches. While there are plenty of choices for the quantification of reduced thiols, much fewer techniques exist for the measurement of oxidized thiols, in particular at their low natural abundance; however, this is of utmost importance for an accurate assessment of thiol redox equilibria. Given the significance of glutathione as cellular antioxidant the majority of analytical developments revolved around GSH/GSSG. Since the pioneering analytical work of Reed et al. [15] most investigators are aware of the need to prevent thiol oxidation in order to avoid an artificial elevation of disulfide levels. However, not only GSH/GSSG but also the extracellular cysteine/cystine ratio is an important read-out of the whole body redox poise (and apparently uncoupled from the glutathione redox) [1], and the ratio of cystine and glutathione has recently been proposed as a new biomarker for oxidative stress in the cardiovascular disease setting [17]. Yet, an even simpler measurement of *total serum free thiols* by means of Ellman's reagent has been shown to predict mortality in patient cohorts [23], [24]. How the latter relates to glutathione and cysteine redox state is currently unknown. While the complexity of changes in extracellular thiol levels has been recognised and the dynamic interaction between *free reduced* and *oxidized* as well as *protein-bound thiols* been defined as “*plasma redox thiol status*” more than two decades ago [49] the main factors and regulatory element that define these interactions in whole blood remain ill defined. This uncertainty is accompanied by new developments that appear to highlight the involvement of sulfide and associated metabolites such as inorganic and organic persulfides and polysulfides [5], [6], [26]. This interaction gives rise to oxidized thiols in which the sulfur of the SH group of Cys, GSH or sulfide (and likely other thiols) is bound to one or more sulfur atoms; the properties of the resulting thiol derivatives differ fundamentally, both chemically and biologically, from the originating thiols [50], [51].

Moreover, even seemingly well characterized compounds such as glutathione seem to have additional actions beyond those of their classical antioxidant function, which would seem to warrant further exploration [52]. Yet, many pitfalls surrounding its analysis in cells and biofluids (some of which have been known for decades) often appear to have been neglected and remain to be better characterized to avoid the enormous variations in GSH/GSSH ratios reported in the literature [53]. Even sophisticated, validated and robust methods for the simultaneous determination of glutathione and cysteine redox state [54] would seem to leave room for improvement inasmuch as pre-analytical sample handling is somewhat cumbersome and time-consuming, involving multiple steps, and total assay time is rather long by today's standards. More importantly, however, the technique used in this particular case (which is based on the technique established by Reed et al. [15]) is unsuitable for the analysis of thiols that do not also contain an amino group such as sulfide or methanethiol. Hence, novel analytical methods with improved sensitivity and specificity, a simplified workflow and higher speed of analysis without compromising robustness and versatility are required to allow future study of the complex interactions between different elements of the thiol redox metabolome in larger numbers. Herein, we provide one example for how such an approach might be put into action.

### 4.2 Establishing an analytical platform for detection of the redox metabolome

Here we describe the necessary steps needed to establish an analytical platform for high-throughput characterisation of the thiol metabolome in biological matrices. As soon as the analytical instrumentation is available, the first fundamental step to this end is to define the relevant target analytes as well as the internal standards for their quantification; from there on, one can proceed with optimization of detection and separation conditions as well as a critical assessment of linearity, precision, LOD and reproducibility of the method. These steps are clearly dependent on the type of instrumentation available, but in part also on the different chemistry of the analytes intended to be covered. However, the main objective of the present method development was not to identify ideal conditions for analysis, but rather to find conditions that are fit-for-purpose for high-throughput redox profiling of large numbers of samples under everyday conditions. To this end, we found that it was not the instrumentation available but rather the sample preparation that represented the most important factor which defines the qualitative and quantitative results.

We chose human blood as suitable starting material for our development. The reason for this choice was severalfold: not only is it a readily accessible and relatively abundant starting material, but it also represents a sort of systemic equilibration medium that is in contact with all other compartments of the body [3]. The redox buffering capacity of the blood (and in particular of RBCs) is notoriously high, and it appears to be kept constant by multiple enzymatic and non-enzymatic mechanisms [3], [55]. For the same reasons the determination of ‘*redox reserve’* (total thiols) and ‘*redox status*’ (ratio of reduced/oxidized thiols) in blood may be relevant to understand the status of biological fitness and resilience of an individual (or animal) under study.

The first critical steps during development were therefore to find a way to stabilize the sample, to separate cellular components from the plasma without damaging RBCs (which contain millimolar concentrations of GSH [55]), in a quick and straightforward way, compatible with rapid and reproducible collection of clinical specimens in a critical care setting, for example. We found that one of the easiest ways to collect blood in this context was to choose commercial EDTA vacutainers since this anticoagulant is also a metal chelator, which may help avoiding secondary metal-dependent oxidative reactions (such as the Fenton reaction). Reduced thiols in EDTA anticoagulated blood can then be stabilized by treatment with an excess of alkylating agent, added within the first 1–2 min after blood withdrawal. In the present study we used NEM for this purpose, whilst others have used different alkylating agents such as IAM or mBB. We opted for NEM due to its superior speed of reaction with SH groups at physiological pH (see below). Regardless of the nature of alkylating agent employed in this context, we observed that addition of these chemicals to whole blood leads to an artifactual increase in GSH and sulfide concentrations in plasma if centrifugation is carried out at low speed for too long. This problem was circumvented by use of a high-speed/short-centrifugation protocol for cell separation, indicating that this phenomenon was an artefact possibly due to an alkylation-induced increase in RBC membrane permeability. Indeed, NEM is well known to affect RBC membrane properties, inducing a loss of membrane asymmetry and exposure of phophatidylserine as well as to modifying critical thiols in ion channels and transporters, thereby affecting ion exchange processes, decreasing cell deformability and increasing membrane leakage [55].

Another important factor that requires careful consideration in methodological developments is the choice of protein removal method. Biological fluids such as plasma are complex matrices, the constituents of which can potentially cause interference with chromatographic separation and analyte detection by e.g. lowering ionization efficiency or ion suppression. In the present study, biological samples were cleared before analysis, and the majority of proteins were removed by ultrafiltration over a 10 kDa cut-off membrane using centrifugal columns. The classical methods described for determination of aminothiols, specifically GSH, using either chromatographic techniques or enzymatic assay, also normally include a protein removal step. Many standard deproteinization procedures are based on addition of acids (trichloroacetic acid, SSA) or metals like zinc sulfate/NaOH. Clearly, either of these methods would cause drastic changes in pH, which may affect detection of sulfur species and sulfide [27]. In fact, sulfur species are well known to be particularly labile under acidic conditions; on the other hand, sulfur can be released from thiols under alkaline conditions [27], leading to an artificial elevation of sulfide. In addition, zinc sulfate is known to trap free sulfide and form a water-insoluble ZnS precipitate. Alternative protein removal processes include addition of polar organic solvents such as methanol, acetonitrile or acetone, or solid phase extraction. We observed that using organic solvents for protein precipitation decreases the recovery of GSSG; moreover, the high concentration of organic solvent in the sample sometimes interferes with the chromatography (which often requires an almost complete aqueous phase to start with). Therefore, in order to quantify aminothiols and sulfide using an identical sample processing procedure, clearing samples by ultrafiltration, which does neither change the pH nor add any acid or solvent, to us appears to be best practice, at least for matrices such as plasma.

A further important step was to determine the stability of the samples after freeze/thaw. We found that except for NAC (levels of which dropped by 30–50% after freeze/thaw compared to immediate analysis) concentrations of all other thiols did not drop but rather increased in concentrations after freeze/thaw. The reason for this behavior is not immediately obvious, but clearly there is no problem with the stability of the thiol-NEM adduct. We suspect that this observation may be related to the stability of thiol derivatives that previously escaped detection (perhaps a metastable compound that decomposed during the freeze/thaw process such as a persulfide and/or polysulfide). However, levels of sulfide did not change under the same conditions, suggesting that the product of decomposition of these per/polysulfides might be a higher oxidized form not captured by our assay. Interestingly these effects of freeze/thaw were not observed when proteins were removed by addition of SSA. This suggests an alternative explanation that is even more intriguing: conceivably, aminothiols may exist not only in *free* and *protein-bound* form (whereby binding is via a mixed disulfide and therefore DTT-reducible), but a fraction of them also binds to proteins in a non-covalent fashion. When protein conformation is changed by precipitation with acid/organic solvents or as a consequence of a freeze/thaw process this non-covalent association is perturbed, making more aminothiols available for trapping by the excess NEM present. This scenario might also explain the apparent differences in absolute concentration of analytes (which were independent of the detection method) when plasma was processed by ultrafiltration versus acid precipitation; moreover, it might offer an explanation for our observation that not only sulfide, but also the concentration of some aminothiols increases upon acid treatment. The notion of non-covalent protein binding of thiols is also consistent with the observed concentration-dependence of NEM in plasma - why should levels of certain aminothiols (and sulfide) otherwise further increase with higher NEM concentrations unless they are removed from an equilibrium? This concentration-dependent phenomenon was particularly prominent with sulfide (see Fig. 3**E**), and consistent with a recent report that H_2_S non-covalently binds to albumin and hemoglobin [36]. These aspects of the thiol redox metabolome would seem to warrant further investigation as it might offer yet another, hitherto unrecognized level of redox regulation by modulation of protein association.

### 4.3 Choice of alkylating agent - Why NEM?

Many different thiol alkylating agents and strategies have been developed over the years, and compounds used not only differ in chemical structure and reactivity but also in the mechanism by which they interact with thiols. Therefore, selection of the most appropriate compound for thiol derivatization will be determined by the purpose of its use [56], [57], for example whether the goal includes either labelling or blockage of reactive thiol groups in proteins, thiol quantification in biological samples, or the prevention of autoxidation.

NEM is well known for its usefulness in characterizing sulfur metabolism due to its propensity to rapidly react with SH groups [58]. As a result of its reaction with protein thiols and SH-containing cofactors it can inhibit intermediary metabolism, block vesicular transport, activate specific ion channels and interfere with cell replication, for example. Half a century ago, it was also employed for the spectrophotometric measurement of SH groups [59]. NEM and related maleimides have since been widely used for the analysis of thiols, including GSH [60], [61], for the prevention of GSH autoxidation [61] to stabilize human blood for later quantification of thiols/disulfides [62]; even in a similar workflow for sulfur pathway analysis using a different chromatographic separation [63]. It has also been known for some time to react with sulfide [38], [43]. However, to the best of our knowledge this property has not been used for development of a combined method for thiol and sulfide detection using mass spectrometry.

Of note, the compound's specificity for thiols is not absolute. At high concentrations, NEM can also react with amino groups of peptides/proteins and aminothiols [64]. In fact, its specificity for SH groups strongly depends on reaction conditions; the side-reaction with amines does not pose a major problem as long as the pH of the reaction solution is kept near neutral. However, reagent concentrations in excess of those found optimal for the majority of analytes in a specific biological matrix (determined to be 10 mM NEM for plasma in the present study) may lead to lower apparent concentrations of select analytes. Cys is a case in point and has been shown to undergo intramolecular transamidation to form a cyclic reaction product; this reaction can run to completion under alkaline conditions [64], and this may well account for the progressive descrease in apparent Cys levels in Fig. 3C (when increasing NEM concentrations were tested) as well as the drop in reaction yield when pH was changed from 7.4 to 9 (Fig. 2**B**). Yet, apart from these examples and in spite of its widespread use as a pharmacological and analytical tool for decades, it is somewhat surprising that the reaction mechanism of NEM with thiols has not been investigated in much more detail (this could change in the near future as the underlying chemistry also forms the basis for numerous applications in materials chemistry including the surface modification of porous polymeric monoliths used in chromatography columns [65], [66]).

In order to appreciate NEM's uniqueness among thiol-reactive compounds and explain some of our experimental findings with different aminothiols and sulfide (as well as polysulfides) it may be informative to briefly review key mechanistic aspects of the chemical reaction. This is of importance since reaction conditions not only affect the stability of NEM and the final reaction product (both of which are subject to alkaline hydrolysis [43], [67], [68], but also speed and nature of the reaction itself as well as its specificity for SH groups over reaction with amines. The pH plays a central role not only for reagent and product stability, but also because it affects, along with the pk_a_ of the SH group, the amount of thiolate available. It is a fortunate coincidence for analytical chemistry that the reaction of NEM with thiols proceeds optimally around the physiological pH, i.e. in the range of 7–7.5.

**Scheme 1:**
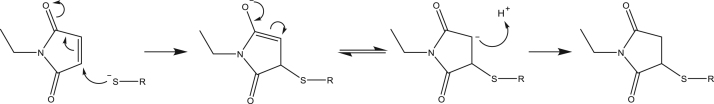
**Mechanism of the reaction between N-ethylmaleimide and thiols (RSH) in aqueous solution.** R- alkyl chain with additional functional groups (as in aminothiols), hydrogen (as for sulfide) or sulfur chains of various length (as in polysulfides).

NEM and related maleimides are electron-deficient compounds (electrophiles) that participate in chemical reactions by accepting an electron pair of a nucleophilic compound such as a thiol. As a maleic acid derivative it also has an activated double bond with which thiols can react. The pH-dependence of NEM's reaction with GSH and Cys [43], [69] indicated that only the thiolate (RS^-^) and not the undissociated thiol (RSH) reacts with NEM. The mechanism involves a nucleophilic addition of the thiolate (a strong nucleophile) onto the olefinic (C˭C) bond of the maleimide ring, forming an intermediate carbon-centered anion; as a strong base this immediately abstracts a proton from the conjugate acid (RSH) or from water, yielding the thioether (see Scheme 1). Reactions of a nucleophile with an α,β-unsaturated carbonyl (or another electron-withdrawing group) are known as ‘Michael addition reactions’ of which the thiol-NEM reaction is a special case. Since the sulfur can add to either side of the double bond it gives rise to different diastereomeres (see Fig. 1B), sometimes complicating the chromatographic separation of thiols [41] by leading to double peaks. Formation of the maleimide-thiol adduct was long thought to be irreversible (unless subjected to electrolysis). More recent studies, however, have documented retro reactions to occur even under physiological conditions, explaining e.g. the faster-than-expected cleavage of certain anti-cancer immunoconjugates and PEGylated products in circulation [70]. For analytical purposes, this is not a concern as such reactions typically proceed over many hours/days and require elevated temperatures.

The reaction of NEM with sulfide deserves special attention not only because two molecules of NEM bind to either side of the sulfide-sulfur (Fig. 1B and Table 1), but also because the same reaction can occur with polysulfides (as demonstrated here for hydrogen persulfide; see Section 3.10). The NEM adduct of S_2_^2-^ (Table 1) was found to be unstable and, over time, decomposed to yield additional S-(NEM)_2_. The reason for the instability of this thiol adduct is not known, but may be related to the ease with which sulfide chains can break and/or how favorable e.g. the NEM-S**˙** radical is as a leaving group. Whatever the reason, the instability of S_2_(NEM)_2_ is in line with recent findings by Akaike's group demonstrating that NEM is unsuitable for the determination of cysteinepersulfide/polysulfide concentrations in biological tissues (Suppl Fig. 2 in [71]). Extrapolating those results to hydropolysulfides (S_x_^2-^ where x can be 2–7) one may expect that part of the sulfide detected by NEM in plasma does not originate from free HS^-^ but be derived from organic and inorganic per- and polysulfides. Our comparison with mBB (the current “gold-standard” for sulfide measurement) suggests that this reagent may suffer from the same limitations. Thus, measured sulfide concentrations with these reagents may represent the sum of free HS^-^ and per/polysulfides, and this warrants further investigation. If in addition free sulfide is also in equilibrium with protein associated forms it may be difficult, if not impossible, to determine the concentration of free sulfide in a given biological system – however, the sum of free and non-covalently bound sulfide and polysulfide (or simply “NEM-sensitive sulfide”) may still be a biological meaningful measure.

NEM has a number of advantages over other thiol alkylating compounds. This includes foremost the speed of reaction with thiols, which is considerably faster than that of mBB or IAA/IAM. This makes it ideally suited for the prevention of autoxidation of thiols to their disulfides during sample preparation. Another advantage is that as an uncharged molecule, it easily penetrates cell membranes – a property it shares with mBB and IAM. While this can be an advantage for the analysis of tissue and intracellular biothiols, we here show that the same property can also lead to an overestimation of true plasma levels unless certain sample handling conditions are met. An advantage that is particularly relevant for mass spectrometric detection is the fact that reaction with NEM improves the ionization efficiency of thiols [72], which explains the excellent sensitivities achieved using ESI-MS/MS, for example.

### 4.4 Assessment of free and bound thiols in human plasma and other biofluids; workflow extension to include acid-labile sulfide

Not only can thiols be oxidized (in vivo and artificially during sample handling) to form *symmetric disulfides* (e.g. from GSH, GSSG and from Cys, CysSS), they also engage in the formation of *mixed disulfides* leading to formation of both LMW and protein disulfides. It was proposed that these protein-bound asymmetric disulfides may either represent a post-translational modification or be involved in inter-organ thiol transport [3]. It is important to note that formation of protein-bound asymmetric disulfides may mask detection of a particular thiol, if only *free thiol* concentrations are assessed. Therefore, if determination of the total amount of a specific thiol (like HCys) is required for diagnostic or prognostic purposes, *total (bound+free) thiol* concentrations are determined following reduction of the covalent bond between the protein thiols and the LMW thiol. Indeed, determination of total HCys was demonstrated to be more predictive of certain pathophysiologies as compared to *free HCys* [73]. For this purpose we subjected the biological samples tested (plasma, blood cells, urine, and saliva) to reduction by DTT. To ensure complete reduction of all bound thiols (incl sulfide) we determined that the minimally effective concentration of DTT and incubation time required to achieve the highest maximal concentration of LMW thiols and sulfide in the final reaction solution was 50 mM DTT and 10 min incubation at RT for all biological matrices tested except blood cells. The latter matrix required 100 mM DTT and 30 min incubation for complete reduction, emphasizing the importance to validate each step of the analytical procedure for the biological matrix intended to be investigated. For plasma analysis DTT was added to NEM-stabilized plasma. However, no significant difference in final concentrations of total thiols were found between samples that had been reacted with NEM before reduction and those that were reduced with excess DTT and then reacted with NEM; this demonstrates that, in contrast to alkalinization which leads to hydrolysis and ring-opening of the thioether [67], the thiol-maleimide adducts formed are stable under acidic conditions.

Acid-labile sulfur is found in bacteria, mammalian and plant cells/tissues, and various analytical methods are available for its quantification [74], [75]. However, origin and biological role of this “pool of sulfide” remains enigmatic. While its presence in cells and tissues has been ascribed to metal-sulfide complexes such as iron-sulfur proteins (e.g. aconitase involved in intermediary metabolism) and Fe-S clusters in complexes of the mitochondrial respiratory chain, sources in biofluids are believed to be mainly organic or inorganic per- and polysulfides. Thus, the additional sulfide demonstrated here to be captured as NEM-adduct following sample treatment with acid may originate from a variety of sources including protein-bound and LMW polysulfides. Some but not all of the DTT-reducible thiols may be acid-labile, although these two classes have been shown earlier to be chemically distinct [76].

### 4.5 Assessment of other thiols and sulfur metabolites: versatility, limitations and outlook

Herein we show that this method may easily be adapted to measure additional very different compounds, including CH_3_SH, NAC and CoA. CH_3_SH is a bacterial metabolic product that also arises as a result of methylation of sulfide and methionine transamination in mammalian cells [77], [78], [79], and a human methanethiol oxidase has been identified, which metabolizes CH_3_SH to H_2_O_2_, formaldehyde and sulfide [80]. We found CH_3_SH to be very well retained on the column, eluting between the other thiols and sulfide but to be extremely sensitive to in-source fragmentation. NAC assumes particular importance in bioavailability monitoring following its oral application as antioxidant or as a mucolytic drug. Determination of CoA in plasma and other body fluids may also assume a particular importance for redox-related studies, because it is a quantitatively important thiol involved in intermediary metabolism that engages in S-thiolation reactions in much the same manner as glutathione [81], [82]. We had some technical difficulties with this particular analyte as the NEM adduct of CoA was retained too strongly by the column due to multiple mechanisms of retention, making it impossible to measure circulating plasma concentrations of this important intermediary metabolite along with other thiols and sulfide in the same run. Nevertheless, we are confident this can be resolved using either a different separation column or modified chromatographic conditions, and this is a focus of our ongoing efforts. This method may be also implemented to assess further key intermediates of the methionine recycling and transsulfuration pathway by the addition of further related compounds such as methionine, S-adenosyl-methionine, S-adenosyl-homocysteine and cystathionine to the existing method, eventually enabling to capture the sulfur metabolome in its entirety.

It is important to note that sulfide levels assessed here by NEM derivatisation are higher compared to methods using other protocols employing mBB [33], [35]. In our hands, absolute *“free” sulfide* levels are strictly dependent on the alkylating agent used, and may depend on the chemistry of the reaction of the alkylating agent; very different concentrations in apparent sulfide concentrations were found with aliquots of the same sample using NEM, IAM or mBB. The reason for these differences warrants further elucidation. Of note, other reports demonstrated that e.g. dBB can extract sulfur from other sources [83], and that NEM reacts differently with polysulfides as compared to IAM or IAA [71].

### 4.6 Strengths and limitations

The platform technology we present here for the measurement of reduced and oxidized thiols in combination with sulfide related metabolites appears to be robust, sensitive and versatile. Its strengths include the possibility to measure the thiol redox metabolome including *total* and *free thiols* and their corresponding disulfides as well as sulfide in complex biological matrices; the technique can be easily combined with other compatible analytical procedures for detection of NO metabolites and nitrosospecies, as established by us before [45], [84], using NEM for stabilization of thiols. This may provide a deeper understanding on the systemic thiol redox state within the RSI. We made an effort to simplify workflows for sample preparation and storage, realizing there will be a need to translate critical steps into easy-to-follow standard operating procedures, in particular when measurements have to be carried out in large numbers, human studies are run in a multi-center fashion and/or biobanked samples are used. Lack of reproducibility and robustness or a very particular sample handling requiring specialist training could easily make a particular method wrongly appear unsuitable for use in clinical studies, or worse perhaps, lead clinicians towards incorrect treatment decisions.

Despite careful optimization, some technical limitations remain. In the current configuration these relate to the relatively poor retention of CysSS on the column, which together with an unknown and variable interference from plasma constituents causes ionization quenching, leading to an underestimation of the true concentration of this disulfide in plasma. This appears less of a problem (or no problem at all) in other biological matrices such as RBCs and murine tissues and may be partially overcome by using the standard addition method to determine absolute concentrations. Moreover, as discussed above, sulfide levels appear higher than with other methods, calling for a systematic comparison of the leading methods described for sulfide assessement [27], [31].

Furthermore, there may be a need for additional methods to cover the per- and polysulfides as important compounds bridging aminothiol and sulfide chemistry. As discussed above, a major limitation of the application of NEM is the fact that it cannot be used for detection of per- and polysulfides, for which the use of β-(4-hydroxyphenyl)ethyl iodoacetamide was carefully optimized and successfully applied in cells and tissues by the group of Akaike and co-workers [71]. The inclusion of these compounds for assessment of the redox metabolome would seem to be a sensible complementary approach to the analytical platform we here introduced.

### 4.7 Summary and perspective: main lessons learned from this development

We have outlined a novel specific, highly sensitive and robust mass spectrometry-based approach for quantification of the thiol redox metabolome. During the development of this analytical approach, we realized that the majority of variability that can be appreciated in the literature assessing aminothiols (and even more in the literature describing the methods to measure sulfide) originates from sample processing procedures rather than the actual analytical method itself. The use of authentic standards and stable isotope labelled internal standards (which are readily available for most aminothiols and sulfide) allow optimization of these methods with limited effort. The choice of analytes to be measured and the availability of the standards should drive the optimization procedures. However, procedures of sample stabilization, for cellular separation and/or isolation (including centrifugation and eventually tissue digestion with detergents), conservation and freeze/thaw cycles all profoundly affect the results produced and therefore also require careful optimization for each biological matrix of interest. Although the analytical approach with NEM presented here is not suited to cover the per- and polysulfide interface, the principles described here may be applied to find an approach allowing detection of an even broader spectrum of metabolites belonging to the thiol redox metabolome. In conclusion, these principles can be applied to assess the thiol redox metabolome in patient cohorts and animal models of disease, thus allowing the identification of the regulatory nodes of redox signaling, while enabling diagnosis, risk assesment, patient stratification and prognosis of redox diseases.

## Footnotes

2 Hydrogen sulfide (H_2_S) in aqueous solution undergoes rapid ionization and is mainly found in the form of HS^-^ with negligible amounts of S^2−^ at pH 7.4. For simplicity, the sum of all three species is referred to as “sulfide” hereinafter.
